# Supplementary material for: Accounting for the long-distance transmission route: An epidemiological model of airborne disease transmission in hospitals
Source: PLoS Comput Biol. 2026 Aug 3;22(8):e1014564. doi: 10.1371/journal.pcbi.1014564 (PMC13460748; doi:10.1371/journal.pcbi.1014564)
Supplement: Supplementary file 1 [file pcbi.1014564.s001.pdf]

Supporting Information

Table of Contents

1. Close-proximity interactions data from the Nods-Cov-2 study....2

a. Description of the participants....2

b. Comparison of the observed and synthetic temporal networks....3

2. Location reconstruction....5

a. Algorithm to reconstruct individual locations based on their category and interactions....5

b. Comparison of reconstructed locations between synthetic and observed temporal networks....7

3. Simulation study....7

a. Grid search procedure to select parameter values for the transmission scenarios....7

b. Correspondence between transmission rates and the basic reproduction number....8

c. Decomposition of short-range transmission in the baseline scenario without intervention....9

d. Comparison of intervention effectiveness....10

e. Sensitivity analyses in scenarios with interventions....14

4. Influenza virus case study....17

5. References....21

1. Close-proximity interactions data from the Nods-Cov-2 study

a. Description of the participants

We apply our simulation procedure to close-proximity interactions data of two adult French ICUs from the Nods-Cov-2 study (1), hereafter denoted as ICU1 and ICU2. Patients, HCWs, administration staff, logistic staff, and visitors who were present at the time of the Nods-Cov-2 study were equipped with wearable sensors recording interactions of less than 1.5 meters over a 36-hour period between April and June 2020. Recording was performed at the start of the COVID-19 pandemic in France, when the historical SARS-CoV-2 variant was circulating. Importantly, this period coincides with strong infection prevention and control measures in hospitals, notably visits from patient relatives were strongly restricted. Ward organization may not be completely representative of non-pandemic times.

In Table A, we detail the number of participants per ICU. Importantly, the number of paramedical staff is much higher in ICU1 compared to ICU2, despite both ICUs having a similar number of patients. We restrict our analysis to patients and HCWs which simplifies the reconstruction of individual locations. Finally, *n*=2 and *n*=2 participants had no interaction recorded over the study period in ICU1 and ICU2, respectively (Table B).

Table A. Number of participants in the two adult intensive care units of the Nods-Cov-2 study. In the main analysis, we focused on patients and healthcare workers, thus we excluded administration staff, logistics staff, visitors, and participants of unknown category in the main analysis.

		**ICU1**	**ICU2**
**Patients**		18	17
**Healthcare workers**			
	Medical staff	30	29
	Paramedical staff	118	46
**Other**			
	Administration	10	6
	Logistics	1	10
	Visitor	1	0
	Unknown	16	4
**Total**		194	112

Table B. Number of individuals without interaction over the study period.

	ICU1	ICU2
Medical staff	2	1
Paramedical staff	0	1

b. Comparison of the observed and synthetic temporal networks

Due to the short time window of the close-proximity interactions recorded as of the Nods-Cov-2 study, the number of patients is too small to simulate realistic outbreaks over 90 days. Thus, we augment the number of patients to reproduce realistic hospitalization stays in French ICUs. We then use a previously published algorithm (2) to generate synthetic temporal networks between HCWs and patients (observed and augmented). This algorithm stochastically generates contacts between individuals using the empirical distribution of contacts between pairs of individual categories (HCW-HCW, patient-patient, and HCW-patient) per hour of the day. This algorithm allows the reconstruction of contacts for individuals that do not have contacts in the observed temporal networks and the augmented patients. Finally, we truncate interactions to match the schedule of the HCWs and hospitalization stays of patients.

In Fig A, we compare (*i*) the dynamics of the number of unique contacts per pair of individual categories and (*ii*) the distribution of contact durations in the observed temporal network and 10 examples of synthetic temporal networks. Synthetic temporal networks reproduce well interactions between individuals as well as their duration.

In Fig B, we compare network properties of the observed temporal network and the synthetic temporal network used in the main analysis. This comparison highlights that the algorithm does not necessarily reconstruct faithfully the same properties across observed networks. Here, we obtain consistent degrees, densities and efficiencies for ICU1, while transitivity (density of triangles) is best reproduced in ICU2.

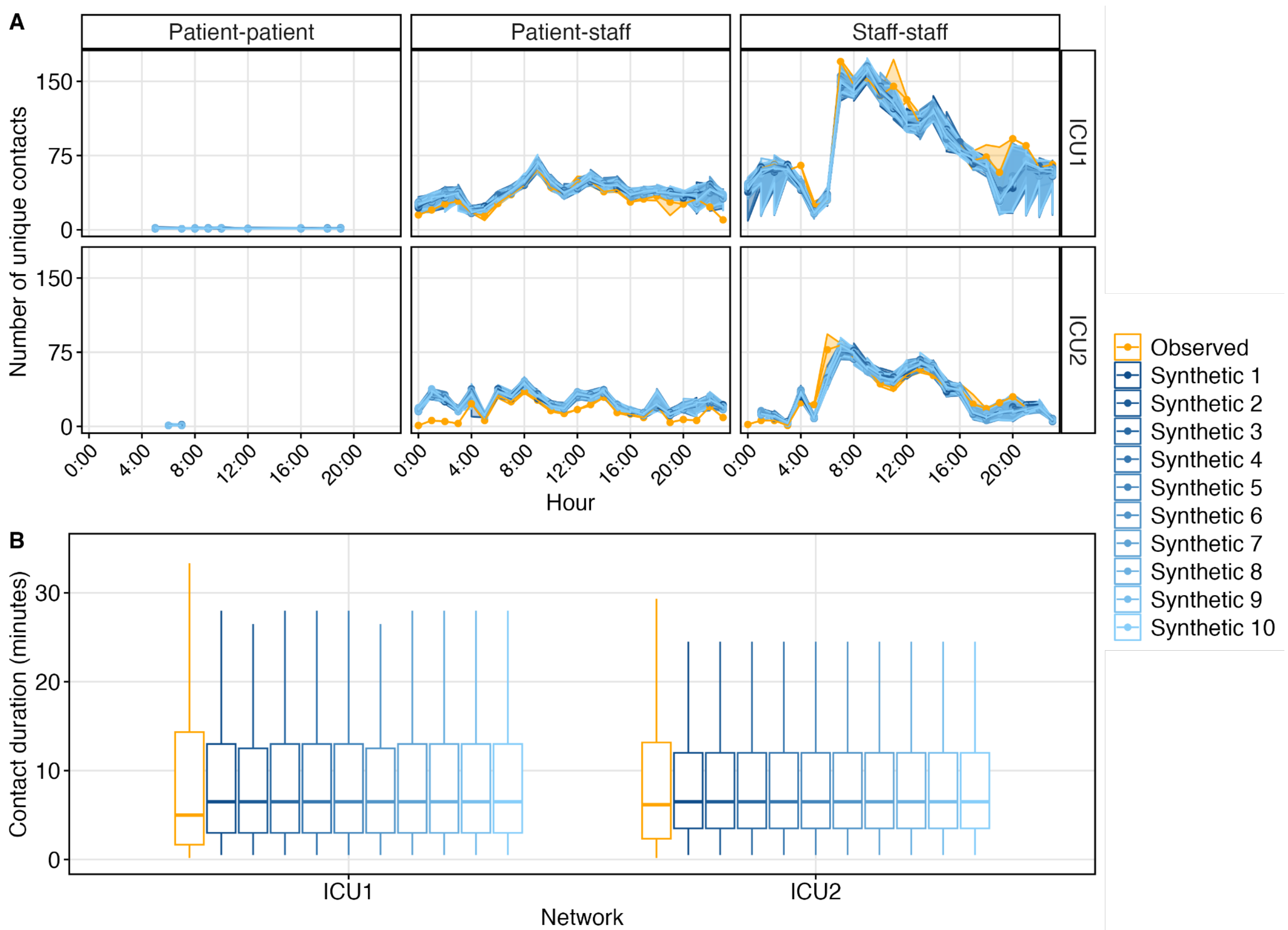

Fig A. Contact patterns of observed and synthetic temporal networks in ICU1 and ICU2. The temporal network reconstruction algorithm is stochastic. We provide here a comparison between ten examples of reconstructed temporal networks (shades of blue) and the corresponding observed temporal network (orange). (**A**) Number of unique contacts per hour in the observed and synthetic temporal networks stratified by pairs of individuals. The solid line corresponds to the median number of unique contacts and the area to the 25% and 75% percentiles. (**B**) Distribution of contact durations (in minutes) in the observed and synthetic temporal networks.

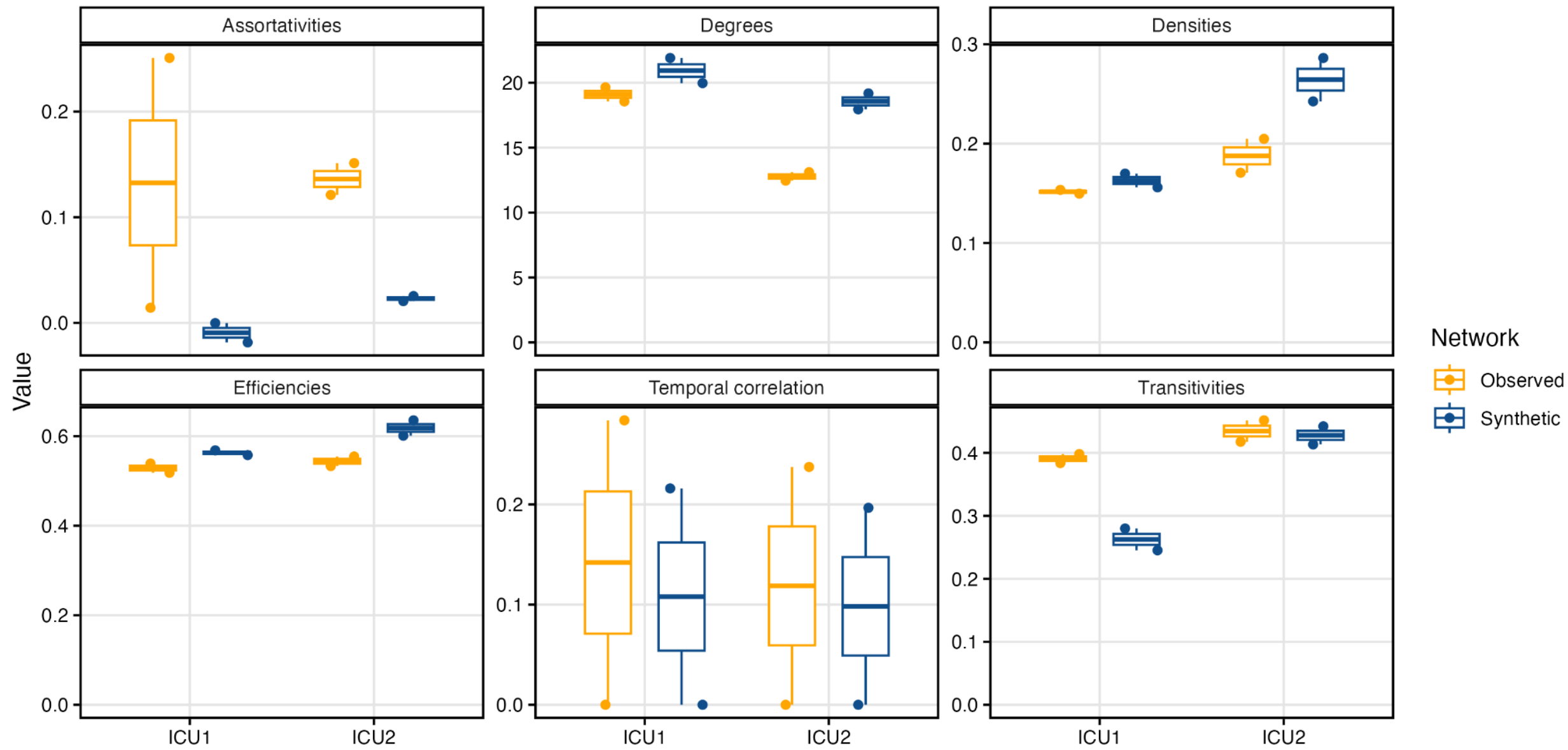

Fig B. Comparison of network metrics for the observed and synthetic temporal networks in ICU1 and ICU2. We compare network properties of the observed temporal networks (in orange) and the synthetic temporal networks (in blue) used in the epidemic simulation study. The two points correspond to the two days of recording in the observed networks and the first two days of the reconstruction in the synthetic networks. The assortativity corresponds to the degree to which similar vertices (i.e. vertices of the same category among patients, medical staff, paramedical staff) tend to connect to each other. The degree is the mean number of contacts. The density is the proportion of edges among all potential edges in the network. The efficiency is the average of the inverse distances between all pairs of vertices. The temporal correlation is the average probability that between two subsequent days, an individual maintains the same number of unique contacts with the same individuals. The transitivity is the probability that adjacent vertices of a vertex are connected.

2. Location reconstruction

a. Algorithm to reconstruct individual locations based on their category and interactions

Our transmission model describes infection due to the exposure to infectious aerosols in rooms that are visited by patients and HCWs. Due to the absence of location data at fine temporal scales, we develop a location reconstruction procedure that reconstructs location of individuals in a minimal ward configuration. This minimal ward is composed of patient rooms, a nursing station, a medical office, a room for the medical staff, a room for the paramedical staff, and a corridor that connects all the rooms. This configuration is applied to both ICUs and does not aim to accurately describe the specific contexts of ICU1 and ICU2. Here, we intend to develop a generic tool that relies on few assumptions, and remains flexible enough to apply to different contexts. Nevertheless, we adapt the volume of the different rooms so that the minimal volume per individual is the same in both ICUs.

The location reconstruction procedure is divided into three steps, as follows:

1. Location assignment during close-proximity interactions (panel B in Fig C): locations depend on the individual's category and the composition of the interaction cluster they belong to. Importantly,

individuals within the same cluster are not necessarily in interaction with all the other members of the cluster. For example, multiple HCWs may be in contact with the same patient but not on the same side of the patient's bed, thus separated by more than 1.5 meters.

2. Individual location smoothing (panel C in Fig C): in this step, we assume that an individual remains in the same room if there is a gap of less than five minutes in a sequence within the same room.
3. Location assignment when individuals are not in interaction (panel D in Fig C): HCWs with no interaction for more than 30 minutes are assumed to be out of the ICU, while patients remain in their room.

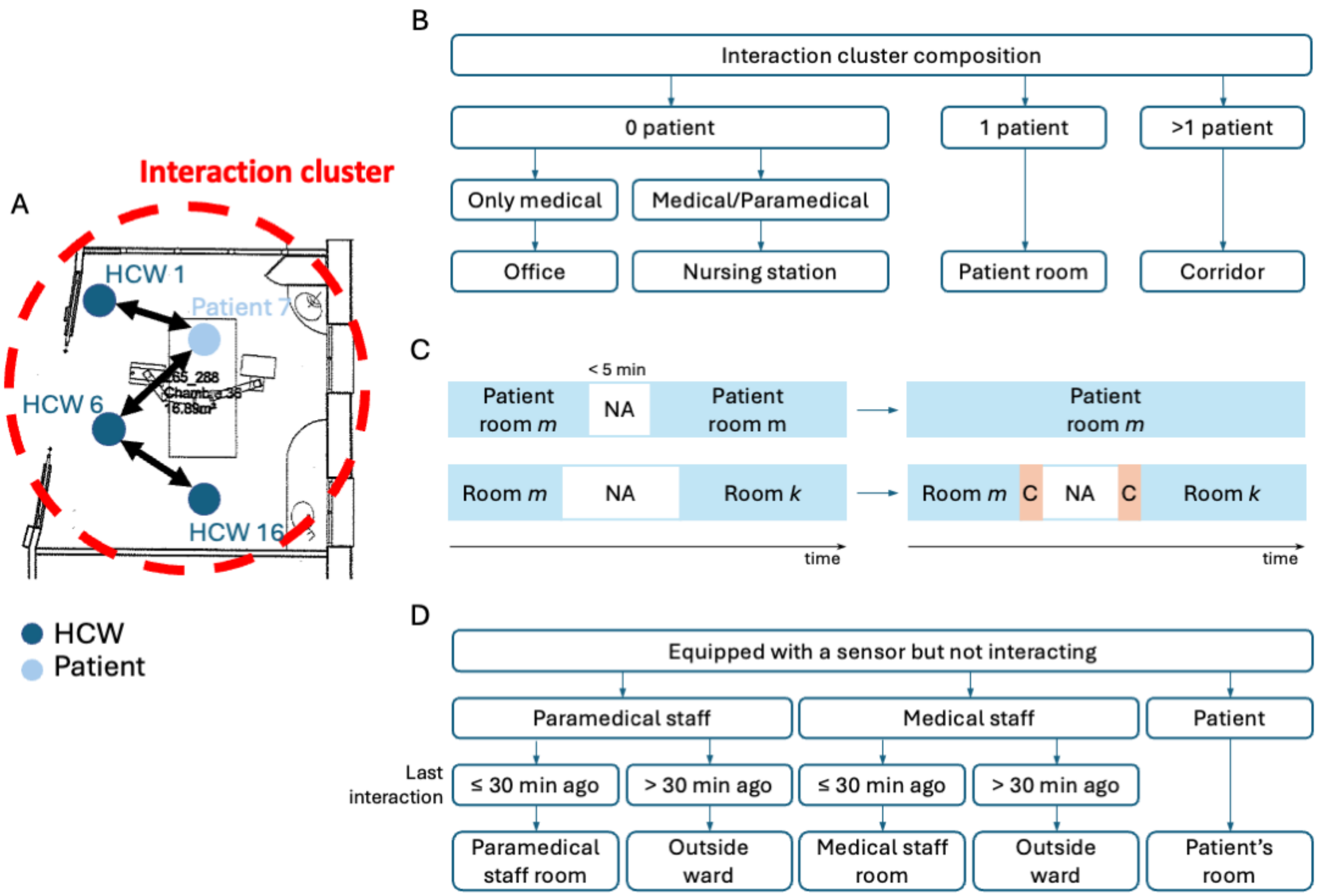

Fig C. Location reconstruction procedure. (**A**) Example of an interaction cluster. (**B**) First step of the location reconstruction procedure. In this step, we reconstructed the location of individuals when they are interacting. (**C**) Second step of the location reconstruction procedure which consists in smoothing spatial trajectories. (**D**) In the third step of the location reconstruction procedure, we reconstructed the location of individuals when they are within the ward but not interacting.

S1 Movie. (separate file)

Example of movements reconstructed for patients and healthcare workers present in ICU1 during the first 24 hours of the synthetic contact data that are used in the main analysis. The movie shows individual locations every ten minutes, but synthetic data is generated every 30 seconds.

b. Comparison of reconstructed locations between synthetic and observed temporal networks

As presented in Fig B, some network properties are less conserved between the observed and synthetic temporal networks, notably network transitivity for ICU1 and network density for ICU2. This has a direct implication on the reconstruction of individual locations illustrated in Fig D. In synthetic networks, there is a slight difference in the cumulative time spent by medical staff, paramedical staff, and patients in the different rooms of the minimal ward compared to what is observed in the Nods-Cov-2 study. Notably, patients spend more time in the corridor (i.e. in interaction with other patients), compared to the observed data. Nevertheless, simulated individual movements are consistent and our model is able to generate aerosol dynamics in the different rooms through the movement of infectious individuals.

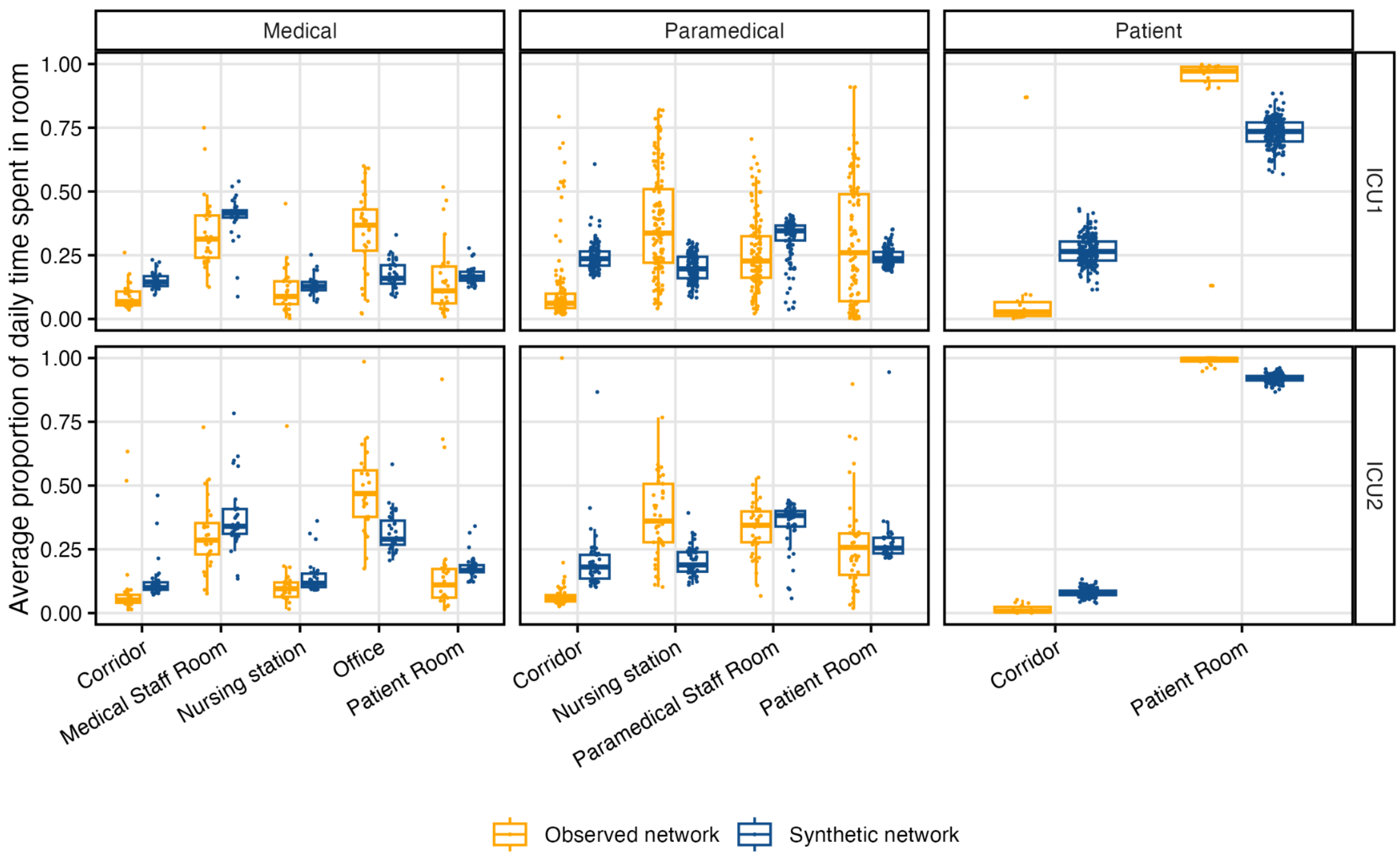

Fig D. Comparison of the proportion of daily time spent in the rooms of the ward between the observed and synthetic temporal networks. For each individual (one circle), we compute the average proportion of the time spent in the different rooms of the ward over their days of presence in the ward. For example, the proportion is averaged over the stay lengths of patients, while it is averaged over the working days for HCWs. This procedure is applied to the observed (in orange) and synthetic (in blue) networks.

3. Simulation study

a. Grid search procedure to select parameter values for the transmission scenarios

We explore value pairs of (β_e, β_c) using a grid search approach and select five value pairs leading to similar secondary attack rates (SAR), defined as the proportion of susceptible individuals that got infected during the 90-day simulation period after the introduction of the index case (Fig E). Selected values are listed in Table 1.

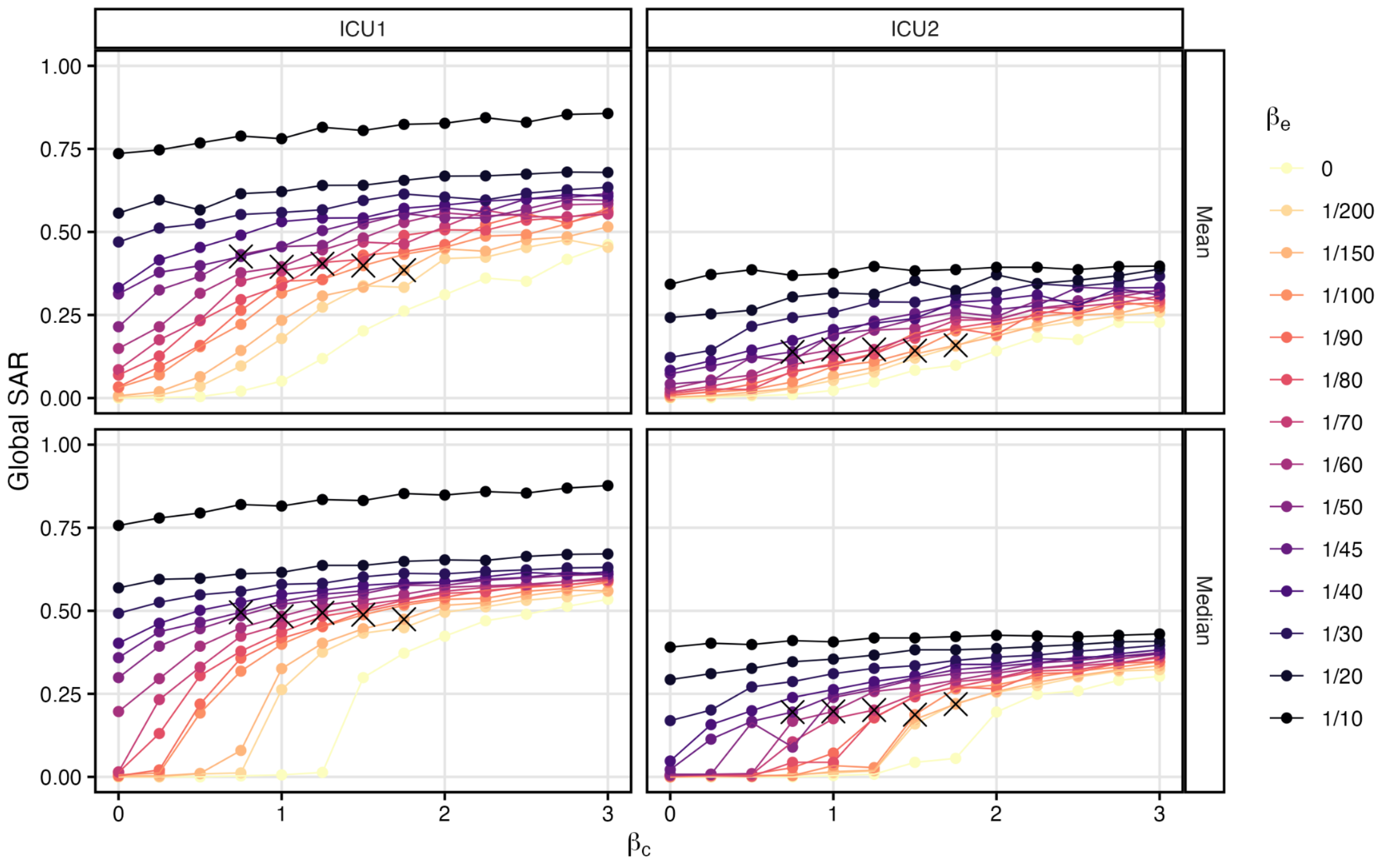

Fig E. Grid search procedure. For each network and value pair of (β_e, β_c), we simulate n=100 epidemics and report the median and average secondary attack rates (SAR). This grid search approach allows us to select five value pairs of (β_e, β_c) depicted with black crosses that represent the five scenarios of transmission explored in the main analysis. These value pairs are chosen so that SARs are similar within each ICU network.

b. Correspondence between transmission rates and the basic reproduction number

Table C. Resulting R_0 for each transmission scheme stratified by category of index case. For each network and transmission scheme, we simulated n=5,000 epidemics over the infectious period of the index case and reported the mean number of secondary cases infected by the index case with its 2.5 and 97.5 percentiles. We stratified the reproduction number by the category of the index case since the medical staff, paramedical staff, and patients have different contact patterns and roaming behavior in the ward, determining the frequency of transmission opportunities. For each simulation, the index case was randomly selected among the individuals present in the ICU the first day of the simulation.

Scheme 1: β_e = 1/45 new cases per infectious aerosol per day and β_c = 0.75 new case per contact per day; Scheme 2: β_e = 1/60 and β_c = 1.00; Scheme 3: β_e = 1/70 and β_c = 1.25; Scheme 4: β_e = 1/100 and β_c = 1.50; and Scheme 5: β_e = 1/150 and β_c = 1.75.

	**Transmission scheme**	**Medical staff**	**Paramedical staff**	**Patient**	**Global**
**ICU1**	Scheme 1	1.57 (1-4)	1.54 (1-4)	2.38 (1-5)	1.64 (1-4)
	Scheme 2	1.56 (1-3)	1.55 (1-4)	2.30 (1-5)	1.64 (1-4)

	Scheme 3	1.58 (1-4)	1.53 (1-4)	2.35 (1-5)	1.63 (1-4)
	Scheme 4	1.60 (1-4)	1.56 (1-4)	2.25 (1-5)	1.64 (1-4)
	Scheme 5	1.57 (1-4)	1.53 (1-4)	2.47 (1-5)	1.65 (1-4)
**ICU2**	Scheme 1	1.42 (1-3)	1.51 (1-4)	1.49 (1-3)	1.48 (1-3)
	Scheme 2	1.45 (1-4)	1.50 (1-3)	1.46 (1-3)	1.48 (1-3)
	Scheme 3	1.42 (1-4)	1.52 (1-4)	1.53 (1-3)	1.49 (1-4)
	Scheme 4	1.44 (1-4)	1.53 (1-4)	1.49 (1-3)	1.49 (1-4)
	Scheme 5	1.44 (1-4)	1.53 (1-4)	1.49 (1-3)	1.50 (1-3)

c. Decomposition of short-range transmission in the baseline scenario without intervention

In all transmission schemes, most of the short-range transmission events occur between HCWs, followed by transmission from HCWs to patients, from patient to HCWs, and finally transmission between patients (Fig F). This is the case in both ICUs which highlights the central role of HCWs in the dynamics of healthcare-acquired infections.

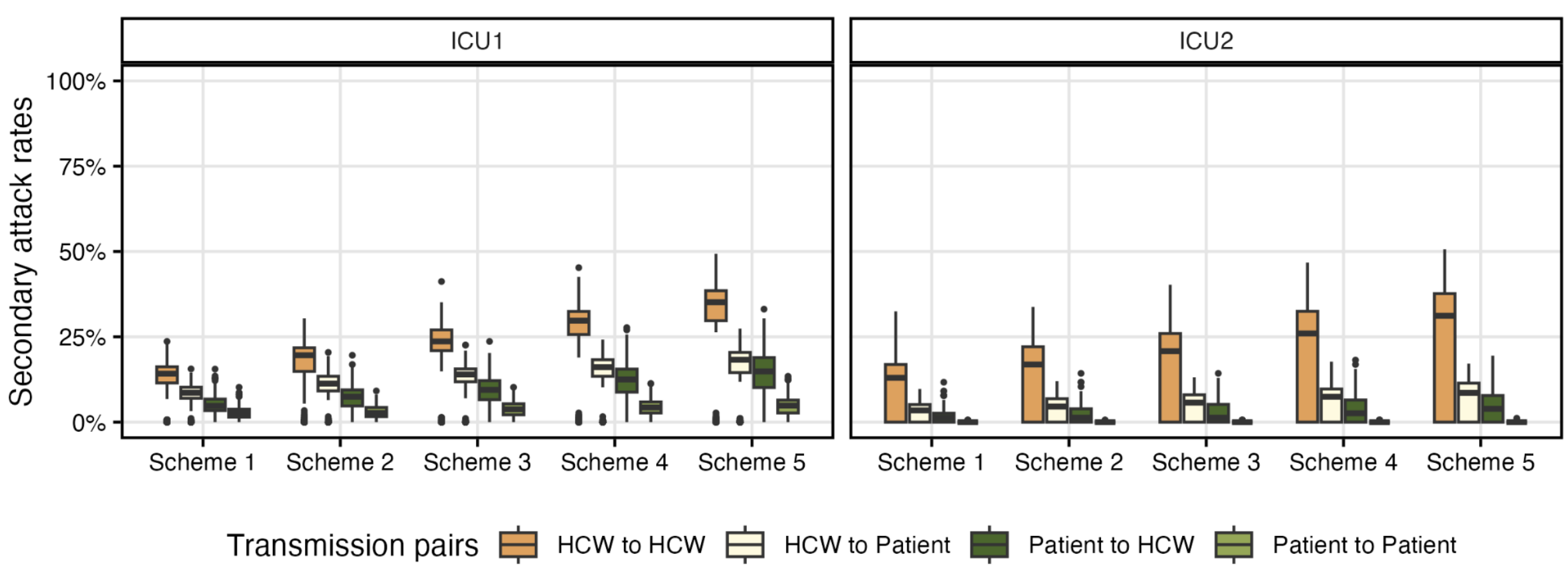

Fig F. Transmission pairs during short-distance transmission events. We report the secondary attack rates among patients or HCWs depending on the category of their infector during short-distance infections. In all transmission scenarios, the highest secondary attack rates are due to infections between HCWs, followed by infections from HCWs to patients, then patients to HCWs and finally patients to patients that is anecdotical in ICU2. As expected, when the short-distance route is predominant, SARs increase for all pairs.

Scheme 1: β_e = 1/45 new cases per infectious aerosol per day and β_c = 0.75 new case per contact per day; Scheme 2: β_e = 1/60 and β_c = 1.00; Scheme 3: β_e = 1/70 and β_c = 1.25; Scheme 4: β_e = 1/100 and β_c = 1.50; and Scheme 5: β_e = 1/150 and β_c = 1.75.

d. Comparison of intervention effectiveness

In Fig G, we present intervention effectiveness in patients, in paramedical staff, and in medical staff. We also report effectiveness among HCWs by pooling paramedical and medical staff, as well as the overall effectiveness, when considering all individuals. Strikingly, only two interventions, universal masking and ventilation in rooms that are inaccessible to patients, reduce transmission among HCWs as much as among patients. Given that HCWs are a major source of infection for patients (Fig F), interventions should also target HCWs. Overall, universal masking is the most effective intervention in both ICUs and across all transmission schemes (Fig H). Improving ventilation in rooms that are inaccessible to patients indirectly contributes to the reduction of short-range transmission by HCWs (Fig I). This is the case in Scheme 1 and Scheme 2 where the long-range transmission is predominant.

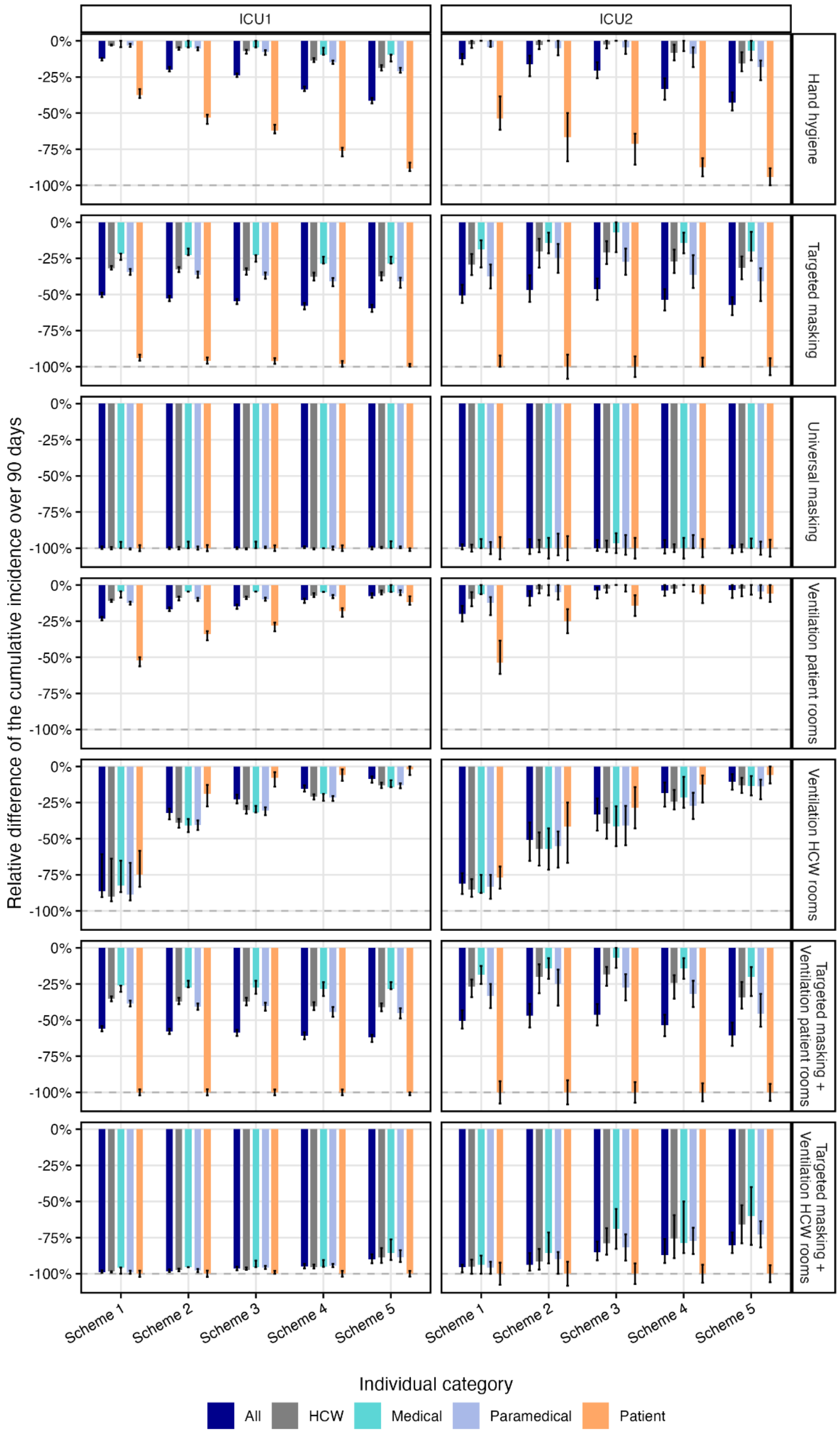

Fig G. Relative difference of the cumulative incidence over 90 days compared to the baseline scenario without intervention and stratified by individual category. Relative difference of the 90-day cumulative incidence among a specific category of individuals between the intervention scenario and the baseline scenario (with no intervention). Negative values indicate a reduction in the cumulative incidence when an

intervention is in place. Relative difference estimates and error bars are derived from the pseudo-median and the 95% CI of Wilcoxon tests, respectively. Reductions stronger than 100% are allowed since we used the median cumulative incidence in the baseline scenario as the reference and the 95% CI bounds of the difference can exceed this median.
Scheme 1: β_e = 1/45 new cases per infectious aerosol per day and β_c = 0.75 new case per contact per day; Scheme 2: β_e = 1/60 and β_c = 1.00; Scheme 3: β_e = 1/70 and β_c = 1.25; Scheme 4: β_e = 1/100 and β_c = 1.50; and Scheme 5: β_e = 1/150 and β_c = 1.75.

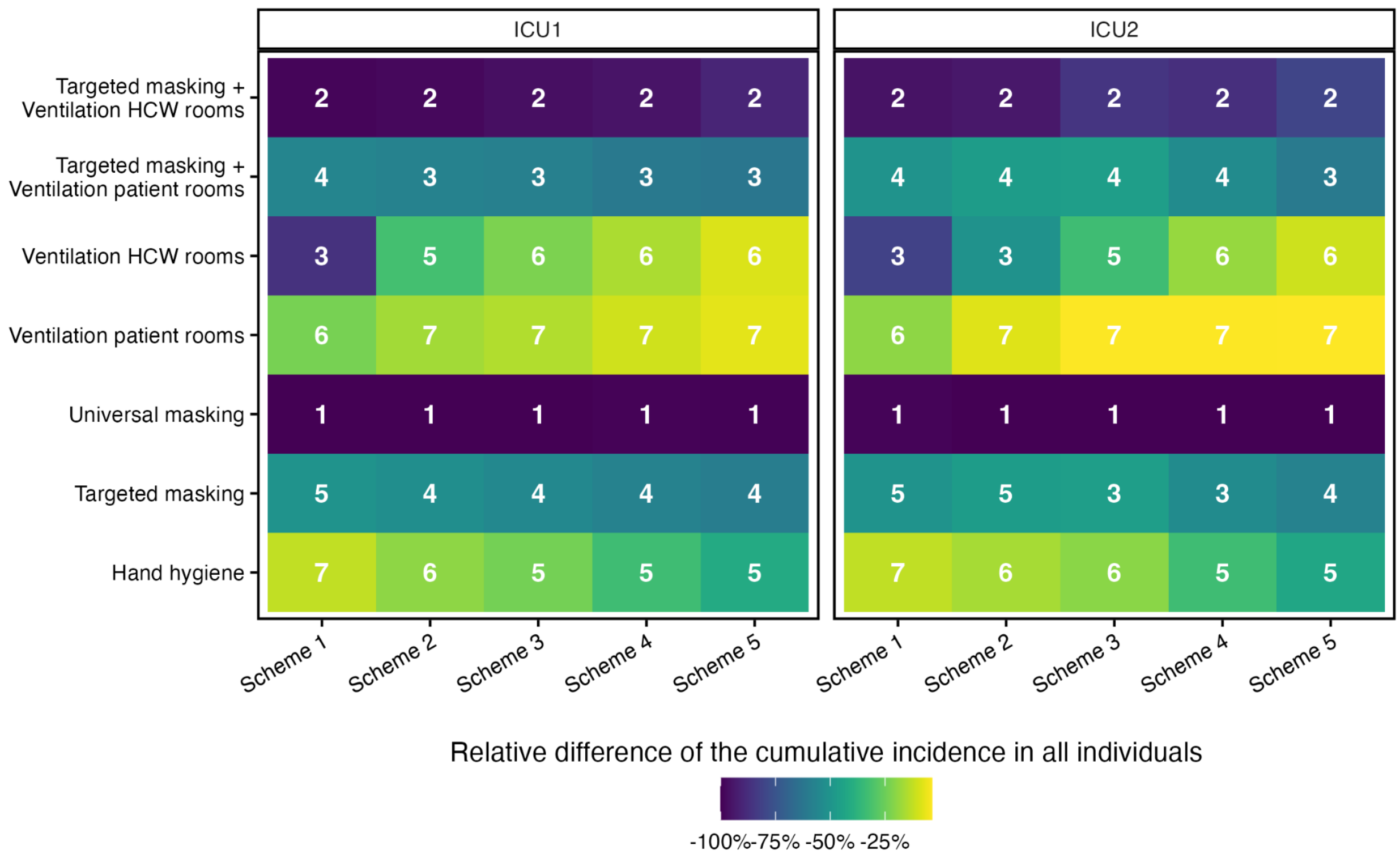

Fig H. Ranking of the most effective intervention in reducing the overall cumulative incidence over 90 days. For each combination of ICU and transmission scheme, we rank the effectiveness of interventions based on the relative difference in the cumulative incidence over 90 days compared to the scenario without intervention. Universal masking emerges as the most effective intervention in both ICUs and across all transmission schemes.
Scheme 1: β_e = 1/45 new cases per infectious aerosol per day and β_c = 0.75 new case per contact per day; Scheme 2: β_e = 1/60 and β_c = 1.00; Scheme 3: β_e = 1/70 and β_c = 1.25; Scheme 4: β_e = 1/100 and β_c = 1.50; and Scheme 5: β_e = 1/150 and β_c = 1.75.

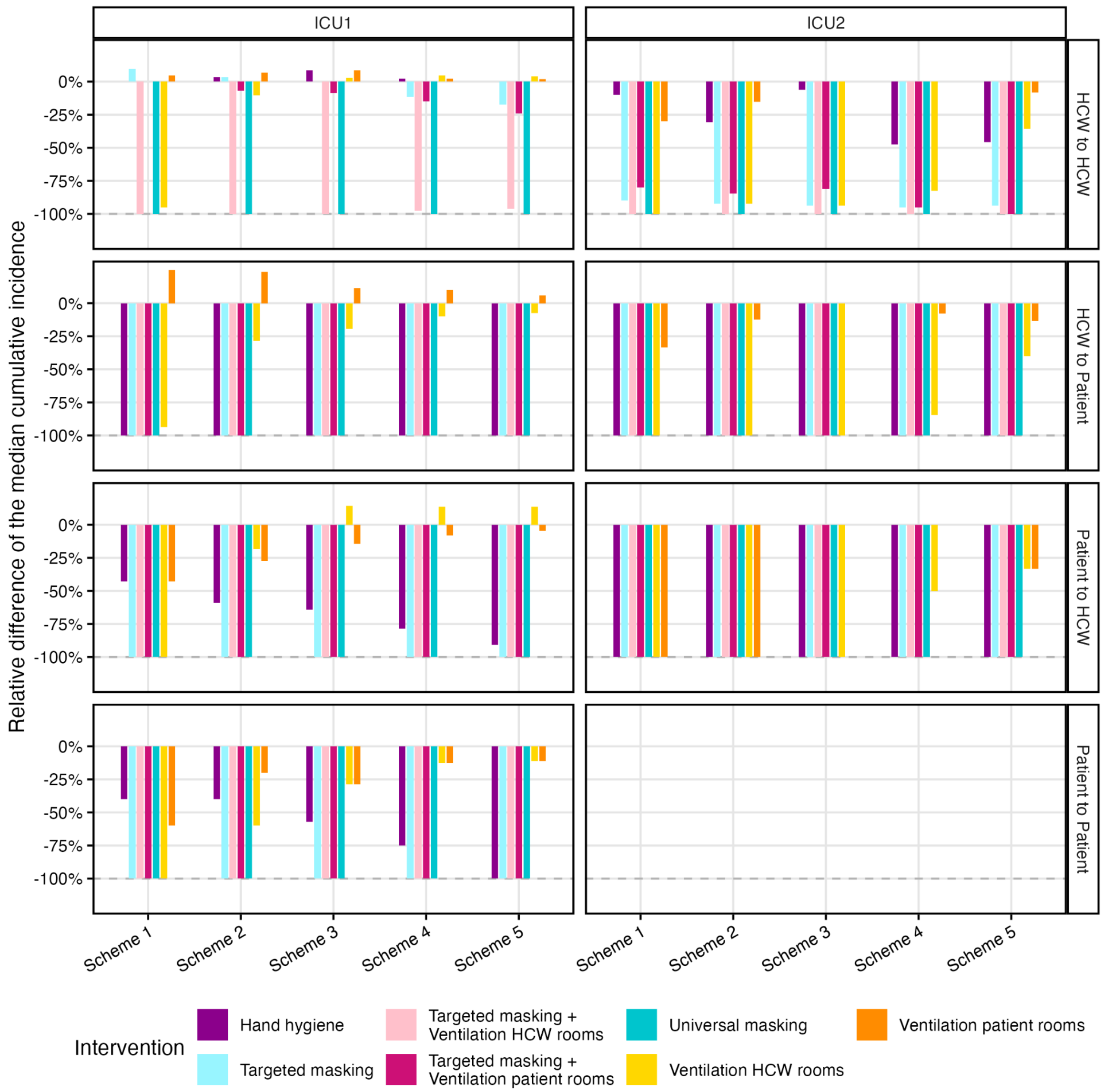

Fig I. Relative difference of the median cumulative incidence for each transmission pair compared to the baseline scenario. Barplots correspond to the relative difference of the median cumulative incidence in the intervention scenario compared to the baseline scenario. In ICU2, the median of the patient-to-patient cumulative incidence is equal to 0 for all transmission scenarios with and without interventions. The relative difference cannot be computed in this specific case.

Scheme 1: β_e = 1/45 new cases per infectious aerosol per day and β_c = 0.75 new case per contact per day; Scheme 2: β_e = 1/60 and β_c = 1.00; Scheme 3: β_e = 1/70 and β_c = 1.25; Scheme 4: β_e = 1/100 and β_c = 1.50; and Scheme 5: β_e = 1/150 and β_c = 1.75.

e. Sensitivity analyses in scenarios with interventions

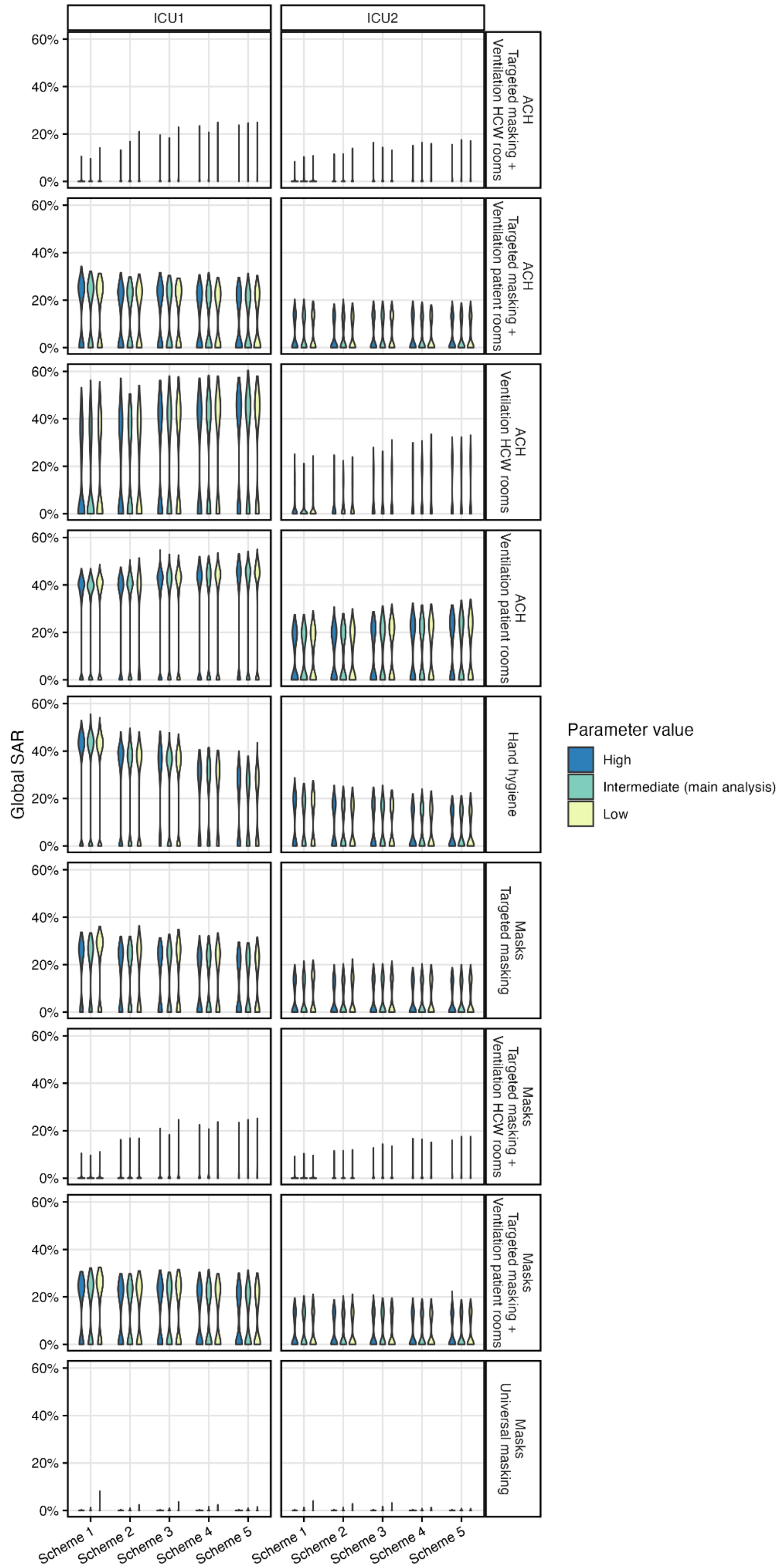

Fig J. Distributions of secondary attack rates in the sensitivity analyses related to intervention effectiveness. We tested low, intermediate, and high values of key parameters governing the effectiveness

of the different interventions (in rows) in a univariate sensitivity analysis across the two ICU networks and the five transmission scenarios. Parameter values are detailed in Table 3.
Scheme 1: β_e = 1/45 new cases per infectious aerosol per day and β_c = 0.75 new case per contact per day; Scheme 2: β_e = 1/60 and β_c = 1.00; Scheme 3: β_e = 1/70 and β_c = 1.25; Scheme 4: β_e = 1/100 and β_c = 1.50; and Scheme 5: β_e = 1/150 and β_c = 1.75.

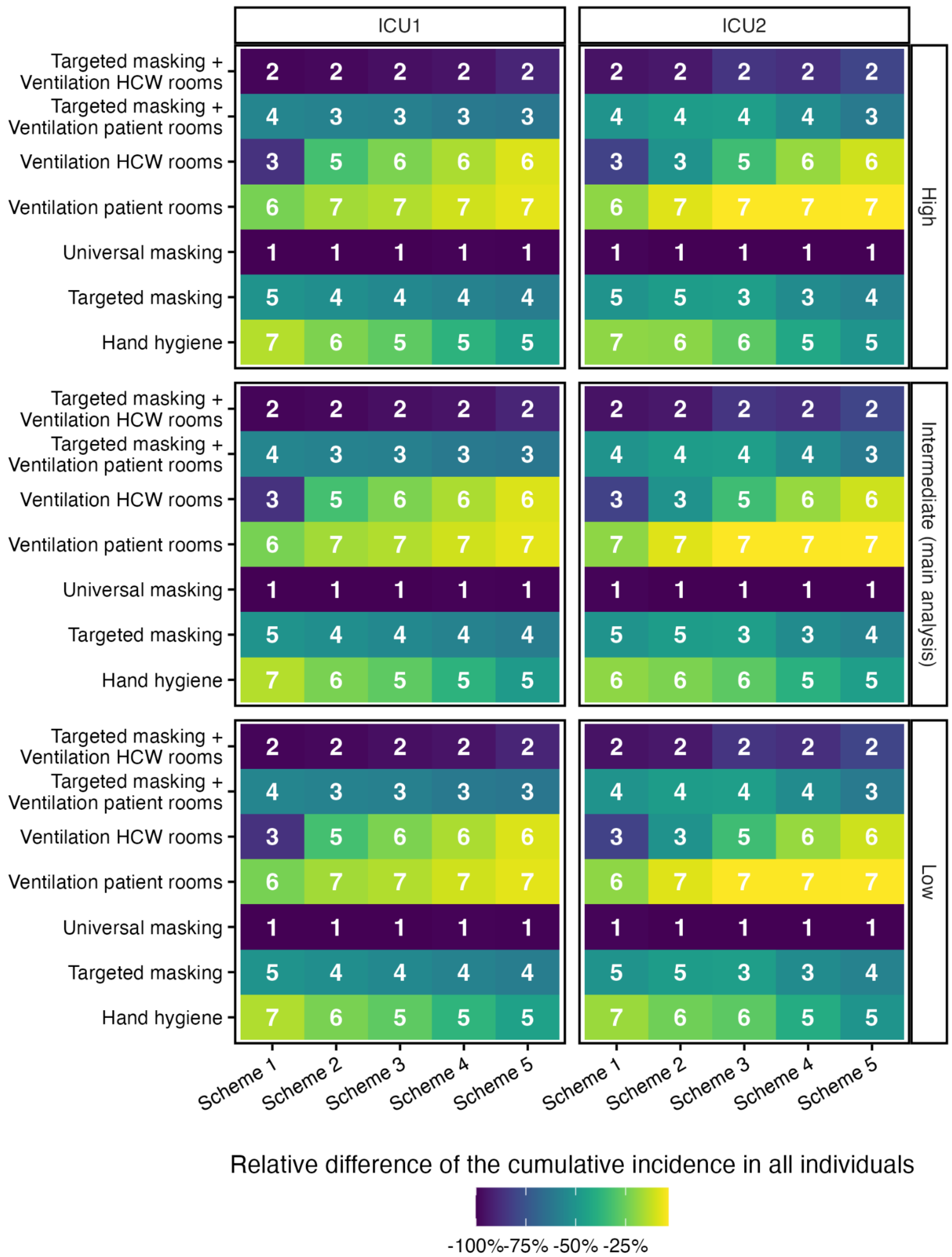

Fig K. Ranking of the most effective interventions in reducing the overall cumulative incidence over 90 days for varying levels of efficacy of hand hygiene.

Scheme 1: β_e = 1/45 new cases per infectious aerosol per day and β_c = 0.75 new case per contact per day; Scheme 2: β_e = 1/60 and β_c = 1.00; Scheme 3: β_e = 1/70 and β_c = 1.25; Scheme 4: β_e = 1/100 and β_c = 1.50; and Scheme 5: β_e = 1/150 and β_c = 1.75.

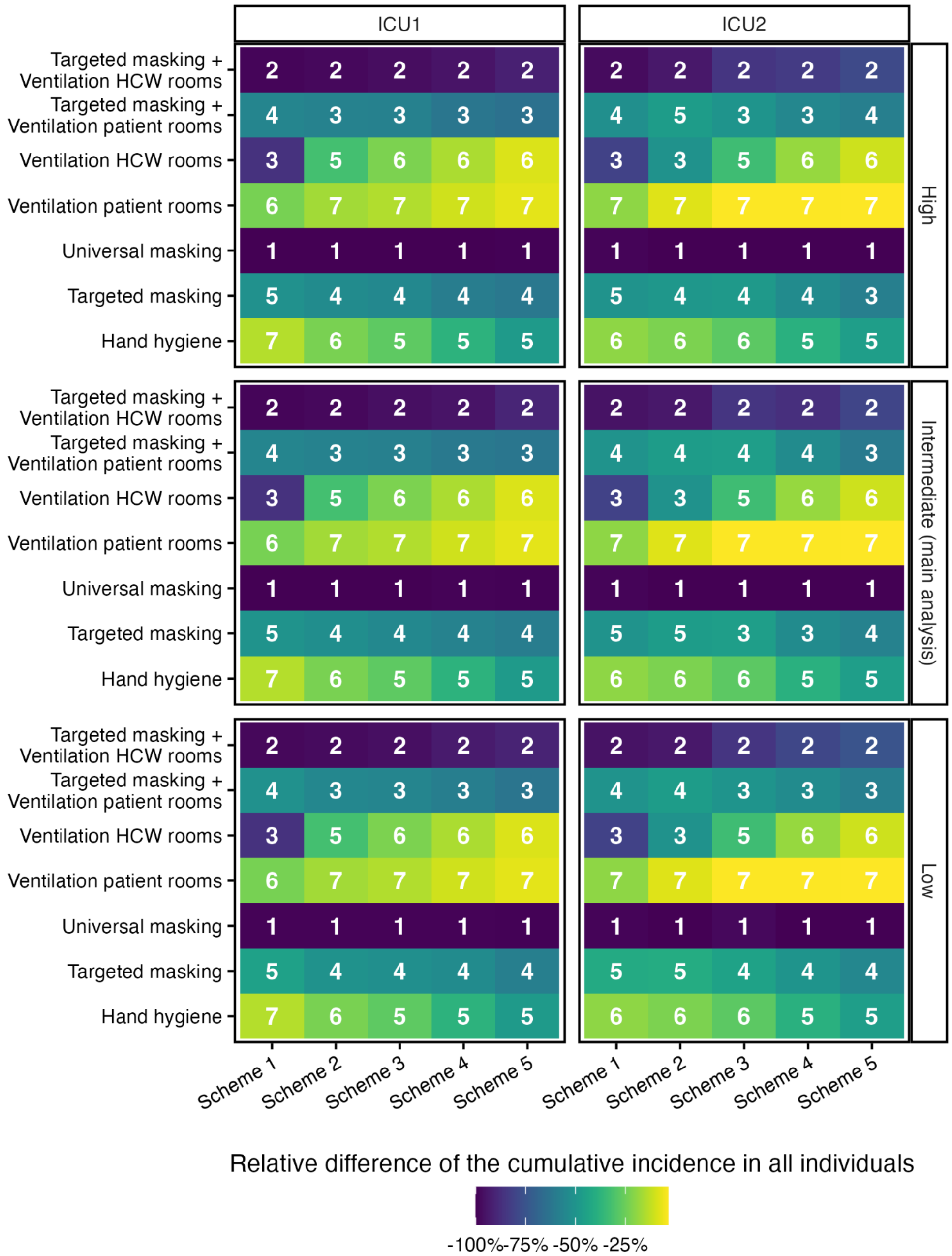

Fig L. Ranking of the most effective interventions in reducing the overall cumulative incidence over 90 days for varying levels of efficacy of mask wearing.

Scheme 1: β_e = 1/45 new cases per infectious aerosol per day and β_c = 0.75 new case per contact per day; Scheme 2: β_e = 1/60 and β_c = 1.00; Scheme 3: β_e = 1/70 and β_c = 1.25; Scheme 4: β_e = 1/100 and β_c = 1.50; and Scheme 5: β_e = 1/150 and β_c = 1.75.

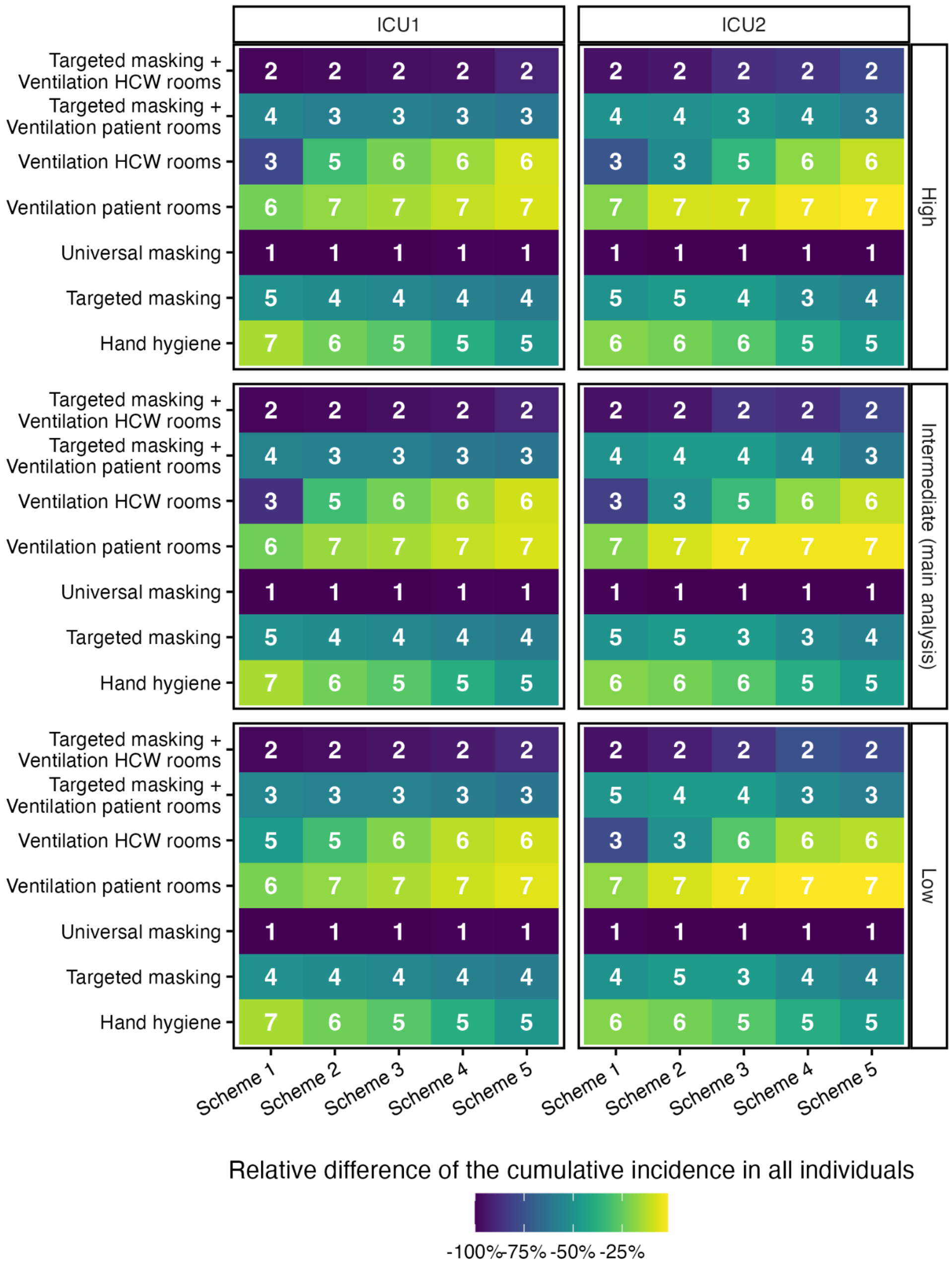

Fig M. Ranking of the most effective interventions in reducing the overall cumulative incidence over 90 days for varying levels of efficacy of ventilation.

Scheme 1: β_e = 1/45 new cases per infectious aerosol per day and β_c = 0.75 new case per contact per day; Scheme 2: β_e = 1/60 and β_c = 1.00; Scheme 3: β_e = 1/70 and β_c = 1.25; Scheme 4: β_e = 1/100 and β_c = 1.50; and Scheme 5: β_e = 1/150 and β_c = 1.75.

4. Influenza virus case study

To further demonstrate the flexibility of our model and the generalizability of our results, we simulated nosocomial outbreaks using parameter values characteristic of influenza viruses (Table D). Given that the importance of the close-proximity route compared with the long-distance route has been little quantified

for influenza viruses, we explored transmission dynamics for the values of the close-proximity transmission rate and the long-distance acquisition rate chosen in the grid search approach for SARS-CoV-2. We informed the other parameters, namely the exhalation rate, the half-life of aerosolized influenza viruses, the incubation period, and the infectious period, from the literature. Importantly, influenza A viruses have been more studied than influenza B viruses, we preferentially chose parameter values of influenza A viruses.

Presymptomatic transmission has been shown to play a role in influenza A virus transmission (3,4). In their modelling study, Zhang et al. (4) estimated that presymptomatic transmission occurred on average 0.7 days before symptoms onset in 16% of flu cases when using the incubation period from the meta-analysis by Lessler and colleagues (5). Thus, we implemented pre-symptomatic transmission similarly to the main analysis on SARS-CoV-2. Presymptomatic transmission occurred in 16% of the cases and could occur at most 0.7 days before symptom onset.

Table D. Parameter values used to parameterize the epidemic simulation model for influenza viruses. The simulation model is parametrized using values taken from the literature, except for β_c and β_e that we calibrate using a grid search approach. Importantly, we inform the inactivation rate of aerosols using the estimates of half-life for seasonal influenza A viruses from Rockey et al. (2024) that lie around 0.55 hours. If we consider that the air of a room is completely renewed when the quantity of aerosols has decreased by more than 99%, then a half-life of 0.55 hours is equivalent to an air change per hour (ACH) of 0.28. Scheme 1: β_e = 1/45 new cases per infectious aerosol per day and β_c = 0.75 new case per contact per day; Scheme 2: β_e = 1/60 and β_c = 1.00; Scheme 3: β_e = 1/70 and β_c = 1.25; Scheme 4: β_e = 1/100 and β_c = 1.50; and Scheme 5: β_e = 1/150 and β_c = 1.75.

Parameter	Description	Value					Unit	Reference
		Scheme 1	Scheme 2	Scheme 3	Scheme 4	Scheme 5		
β_c	Close-proximity transmission rate	0.75	1.00	1.25	1.50	1.75	No. of new cases per contact per day	Grid search
β_e	Long-distance acquisition rate	1/45	1/60	1/70	1/100	1/150	No. of new cases per aerosol per day	Grid search
ν	Exhalation rate of infectious aerosols	2.5e4					RNA copies per 30 min	Yan et al., 2018 (6)
μ	Pathogen-laden aerosol removal due to pathogen inactivation	1.3 (0.55)					Rate per hour (half life in hour)	Rockey et al., 2024 (7)
$\Delta T_{incubation}$	Incubation period	Lognormal(log-mean=0.7, log-sd=0.2)					day	Lessler et al., 2009 (5)
$\Delta T_{infectious}$	Infectious period	U(1,3)					day	Zhang et al., 2026 (4)

In Figs N and O, we present the effects of control interventions on outbreak dynamics of an influenza-like virus. The results are comparable to the SARS-CoV-2 case study with universal masking outperforming the other interventions and targeted masking combined with ventilation in break rooms ranking second. For

some conditions, we observe a limited reduction of incidence in ICU2 that comes from the low transmission levels of the virus in the population, especially in patients and medical staff. Overall, this analysis shows that due to the close natural history between SARS-CoV-2 and influenza viruses, interventions perform similarly across transmission schemes. These results are presumably generalizable to other common respiratory viruses.

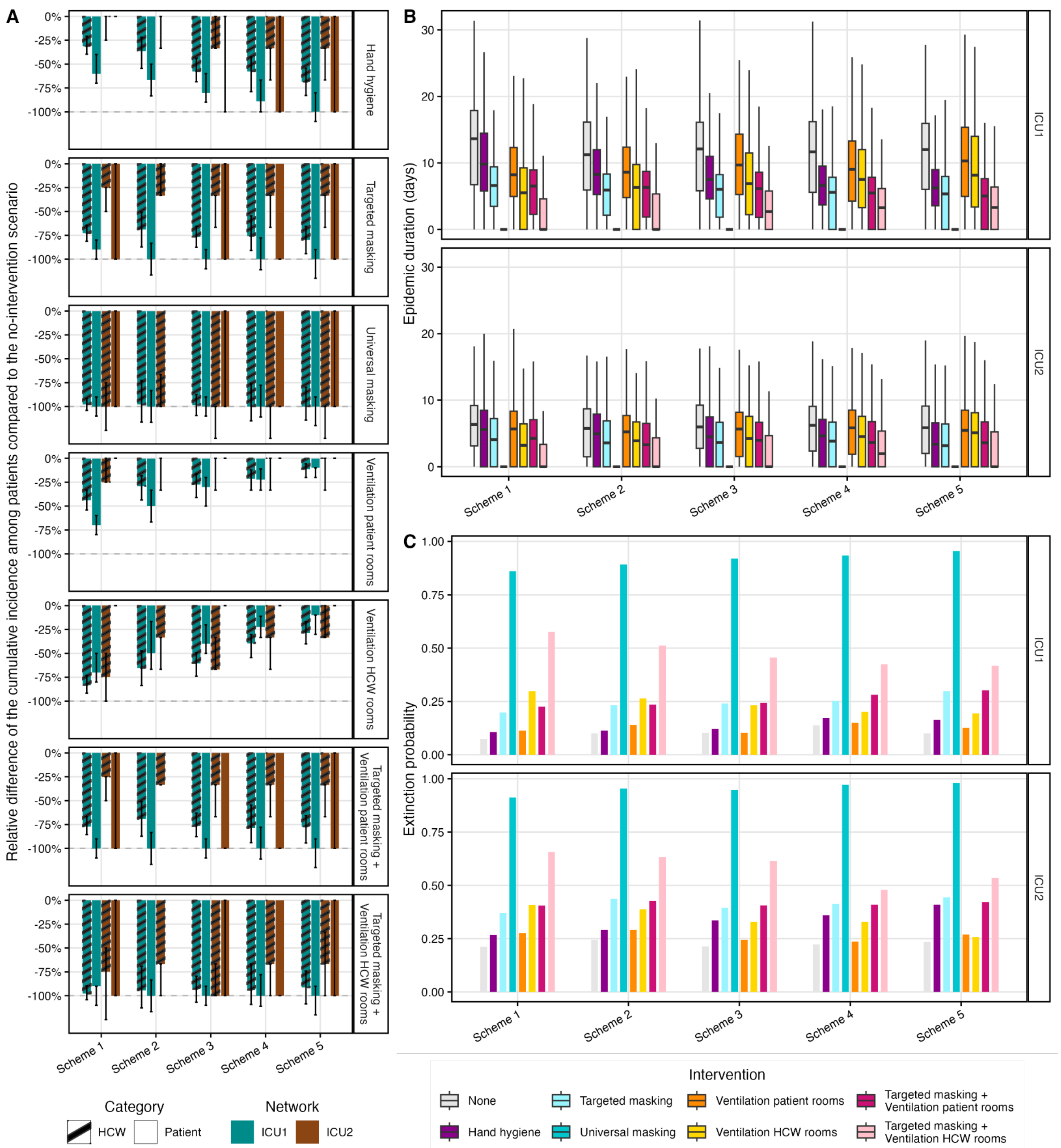

Fig N. Impacts of control interventions on secondary attack rates, epidemic duration, and extinction probability across transmission schemes for influenza viruses. (**A**) Relative difference of the 90-day cumulative incidence among patients and HCWs between the intervention scenario and the baseline scenario (with no intervention). In the hand hygiene scenario, the transmission rate between HCWs and patients was reduced by 14%. In scenarios with targeting masking, transmission rates among patients and between HCWs and patients was reduced by 70% and aerosol exhalation by 94%. In the universal masking

scenario, transmission was reduced by 70% in all pairs of individuals, and aerosol exhalation by 94%. In scenarios with ventilation, we increased ACH to 8. Negative values indicate a reduction in the cumulative incidence when an intervention is in place. Relative difference estimates and error bars are derived from the pseudo-median and the 95% CI of Wilcoxon tests, respectively. Reductions stronger than 100% are explained by the definition of the metric: the median of the cumulative incidence in the baseline scenario used as the reference can be higher than the 95% CI bounds of the difference. Importantly, the median cumulative incidence in patients in ICU2 for Scheme 2 is null when there is no intervention, thus we cannot compute relative reductions in this specific case. Nevertheless, absolute reductions are significantly different from 0 with a p-value < 0.001 for all interventions. (**B**) Distribution of epidemic durations in the baseline and intervention scenarios. (**C**) Extinction probability in the baseline and intervention scenarios.
Scheme 1: β_e = 1/45 new cases per infectious aerosol per day and β_c = 0.75 new case per contact per day; Scheme 2: β_e = 1/60 and β_c = 1.00; Scheme 3: β_e = 1/70 and β_c = 1.25; Scheme 4: β_e = 1/100 and β_c = 1.50; and Scheme 5: β_e = 1/150 and β_c = 1.75.

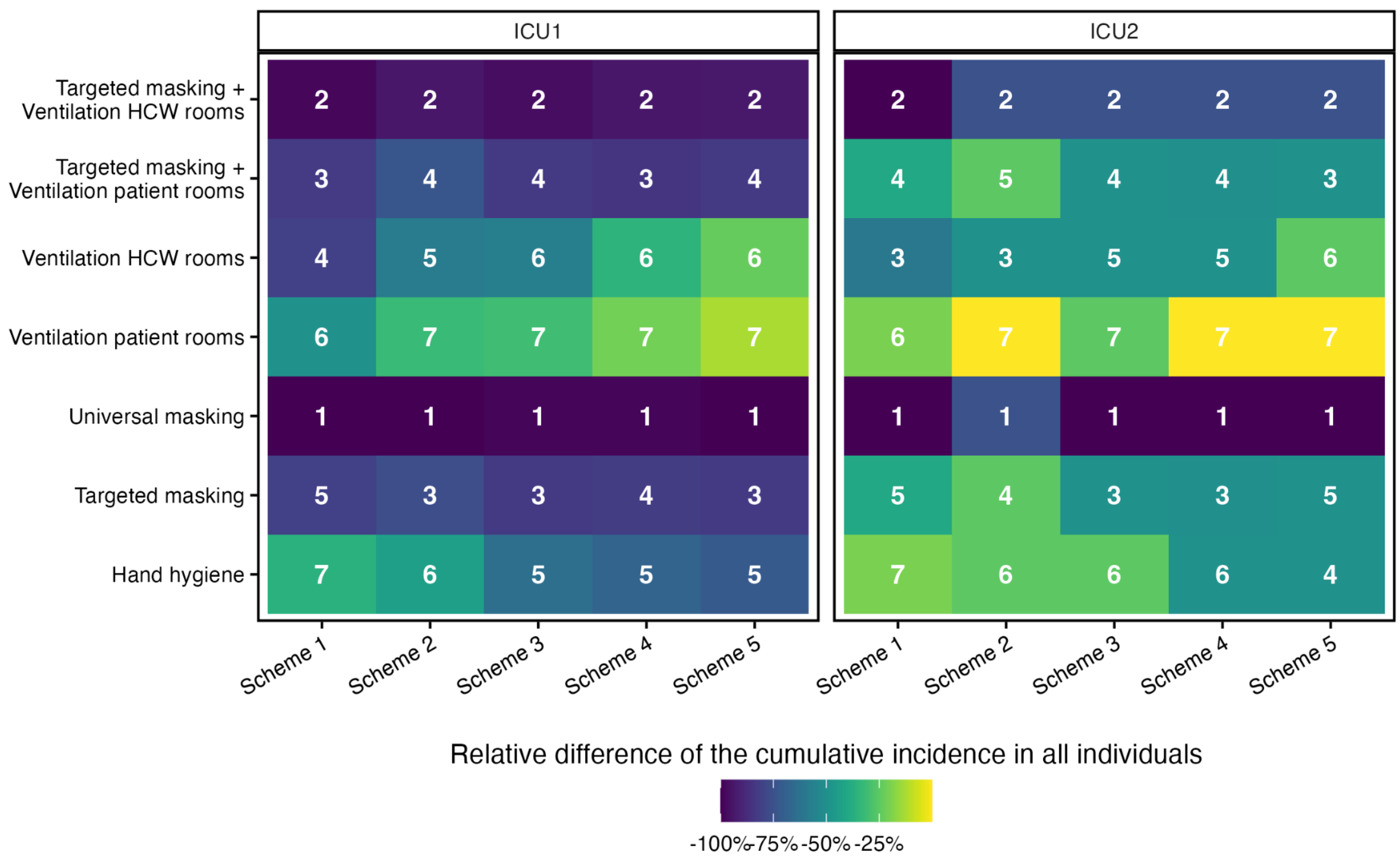

Fig O. Ranking of the most effective intervention against influenza virus transmission in reducing the overall cumulative incidence over 90 days. For each combination of ICU and transmission scheme, we rank the effectiveness of interventions based on the relative difference in the cumulative incidence over 90 days compared to the scenario without intervention.
Scheme 1: β_e = 1/45 new cases per infectious aerosol per day and β_c = 0.75 new case per contact per day; Scheme 2: β_e = 1/60 and β_c = 1.00; Scheme 3: β_e = 1/70 and β_c = 1.25; Scheme 4: β_e = 1/100 and β_c = 1.50; and Scheme 5: β_e = 1/150 and β_c = 1.75.

5. References

1. Shirreff G, Huynh BT, Duval A, Pereira LC, Annane D, Dinh A, et al. Assessing respiratory epidemic potential in French hospitals through collection of close contact data (April–June 2020). Sci Rep. 2024 Feb 14;14(1):3702. doi:10.1038/s41598-023-50228-8

2. Duval A, Leclerc QJ, Guillemot D, Temime L, Opatowski L. An algorithm to build synthetic temporal contact networks based on close-proximity interactions data. PLOS Comput Biol. 2024 Jun 13;20(6):e1012227. doi:10.1371/journal.pcbi.1012227

3. Chan LYH, Morris SE, Stockwell MS, Bowman NM, Asturias E, Rao S, et al. Estimating the generation time for influenza transmission using household data in the United States. Epidemics. 2025 Mar 1;50:100815. doi:10.1016/j.epidem.2025.100815

4. Zhang C, Huang X, Fang VJ, Chan KH, Leung GM, Ip DKM, et al. Frequent presymptomatic household transmission of influenza A but not influenza B virus. Nat Health. 2026 Feb;1(2):206–15. doi:10.1038/s44360-025-00049-y

5. Lessler J, Reich NG, Brookmeyer R, Perl TM, Nelson KE, Cummings DA. Incubation periods of acute respiratory viral infections: a systematic review. Lancet Infect Dis. 2009 May 1;9(5):291–300. doi:10.1016/S1473-3099(09)70069-6

6. Yan J, Grantham M, Pantelic J, Bueno de Mesquita PJ, Albert B, Liu F, et al. Infectious virus in exhaled breath of symptomatic seasonal influenza cases from a college community. Proc Natl Acad Sci. 2018 Jan 30;115(5):1081–6. doi:10.1073/pnas.1716561115

7. Rockey NC, Le Sage V, Marr LC, Lakdawala SS. Seasonal influenza viruses decay more rapidly at intermediate humidity in droplets containing saliva compared to respiratory mucus. Appl Environ Microbiol. 90(2):e02010-23. doi:10.1128/aem.02010-23 PubMed PMID: 38193683; PubMed Central PMCID: PMC10880610.
