# Supplementary material for: Accounting for the long-distance transmission route: An epidemiological model of airborne disease transmission in hospitals
Source: PLoS Comput Biol. 2026 Aug 3;22(8):e1014564. doi: 10.1371/journal.pcbi.1014564 (PMC13460748; doi:10.1371/journal.pcbi.1014564)
Supplement: Supplementary file 2 [file pcbi.1014564.s005.pdf]

Answers to the reviewers' comments

Reviewer 1 1
Reviewer 2 7
Have the authors made all data and (if applicable) computational code underlying the findings in their manuscript fully available? 23
References 24

Reviewer 1

This study uses an agent-based modeling framework to evaluate the roles of close-proximity interactions and aerosol-mediated transmission in healthcare-associated infections (HAIs) in ICUs. The authors use real-world proximity data to generate synthetic contact networks and simulate the transmission of a pathogen with characteristics similar to SARS-CoV-2. They further evaluate several interventions and rank their effectiveness under different transmission scenarios. Aerosol-mediated transmission poses a significant challenge for HAI control, so this study addresses an important and timely question. Overall, the technical approach is sound and the findings are clearly presented. Below are several comments and questions that I hope will help further improve the manuscript.

Response: We thank Reviewer 1 for this positive appreciation.

1. In the simulation design, pathogen spread in the ICU is treated as occurring within a closed system by introducing a single infection at the beginning of the simulation. This design overlooks an important component of HAI dynamics—the interaction with the community. This issue is particularly relevant for SARS-CoV-2, which continues to circulate widely in the general population. Healthcare workers (HCWs) interact with family members and other community contacts, and newly admitted patients may introduce infections into the ICU. It seems feasible for the model to incorporate such external introductions without major changes to the model structure.

Response: We thank Reviewer 1 for this interesting point of discussion. Indeed, we compared outbreak dynamics after the introduction of a single index case, whether a patient or a HCW, depending on the importance of the long- and short-range routes. When it comes to intervention evaluation, it would be interesting to evaluate their robustness to multiple introductions. Our analysis, which considers ICUs as closed systems, probably over-estimates the efficacy of each individual intervention. However we do not expect that additional introductions will change the ranking of these interventions. We did not consider them as adding such introduction pressure from the community would mostly add stochasticity and therefore noise to our predictions and estimates. We have added these considerations in the Discussion section, while rearranging the limitations paragraphs to account for the other considerations of Reviewers 1 and 2.

Discussion (lines 339-345): We acknowledge other limitations in our modelling choices that presumably modulate transmission intensity and the efficacy of the tested interventions. First, outbreak dynamics in

each transmission scheme and intervention scenario were derived from the introduction of a single index case in the ward. This might not be realistic as multiple introductions in hospital wards regularly occur during periods of seasonal epidemics of respiratory infections. This assumption was made to reduce the stochasticity and noise in our estimates. It presumably leads to an over-estimation of intervention efficacy, but this should not impact the ranking of the interventions.

2. The contact data were collected over a 36-hour period. This short sampling window may miss systematic temporal patterns in hospital operations, such as weekly variations in patient volume (e.g., lower patient numbers during weekends). It would be helpful to discuss whether this limitation could affect the representativeness of the simulated contact networks and the robustness of the results.

Response: Indeed, the Nods-Cov-2 contact data spans a short time window for ICUs 1 and 2 and does not include weekends. This may have an impact on the representativeness of the number of individuals and of the number and duration of contacts [1]. We do not expect the number of patients to vary between weekdays and weekends in ICUs, especially during the first wave of the SARS-CoV-2 pandemic when ICUs were overloaded. Concerning HCWs, the number of paramedical staff in ICUs is regulated in France: there should be at least two nurses for five patients and one nursing assistant for four patients. Thus, the number of paramedical staff should remain stable at any time of the week. However, the medical staff is generally reduced at night and over weekends. As a consequence, our simulations over-estimate the role of the medical staff in transmission dynamics. Beyond this consideration of differences between weekdays and weekends, the Nods-Cov-2 study took place during the SARS-CoV-2 pandemic, a very peculiar period that has impacted ward organization and activity. We would expect less HCWs and less contacts between HCWs and patients during non-pandemic times. We now discuss in more detail these aspects in the discussion section.

Discussion (lines 377-384): In addition, the empiric proximity data was collected during the Nods-Cov-2 study which took place in the first wave of the COVID-19 pandemic, when patients were more severe and HCWs more numerous. The resulting interaction networks are ward-specific as shown by Shirreff and colleagues [2], and as a consequence, interaction dynamics are not generalizable to other types of wards or outside of pandemic times. Proximity data was also collected during weekdays and over 36 hours. There are generally fewer medical staff over weekends and most medical procedures are usually scheduled over weekdays. As a consequence the model probably over-estimates transmission over weekends.

3. Some details about the simulation setup are unclear. In particular, what time step was used in the simulations (e.g., minutes, hours, or days)? Close contacts can vary substantially in duration. The choice of time step could influence the calculation of the force of infection. For example, if the model was integrated at an hourly time step, how were short contacts lasting only a few minutes (possibly occurring multiple times within an hour) accounted for? Clarification of this issue would help readers better understand the simulation framework.

Response: We thank Reviewer 1 for bringing up this shortcoming. We fully agree that the duration of close interactions, which drives the risk of infection, is highly variable and can range from a few seconds to several hours as shown in Supplementary Figure 1B. Here, we used a 30-second time step in our simulations. This window is larger than the recording time window of the wearable sensors (10 seconds) in

the Nods-CoV-2 study. However, this choice allows us to account for short-time interactions while remaining computationally manageable when simulating epidemics over 90 days. We have now amended the results and methods section to integrate this information.

Results (lines 174-175) : We then generate a synthetic temporal network over the 90-day period for each ICU, using a time step of 30 seconds.

Methods (lines 389-393): We simulate the transmission of a virus after introduction in the ward. Each simulation starts by the introduction of a random index case and runs over 90 days using a time step of 30 seconds. Parameter values, chosen to illustrate SARS-CoV-2 transmission, are listed in Table 1. Of particular note, room volumes are based on a height of 2.5m and are adapted so that the volume per individual is equivalent at maximum capacity in both ICUs.

4. Intervention parameters were selected from the literature. Masking was assumed to reduce transmission rates by 70%, which may be somewhat optimistic. Under this assumption, universal masking appears capable of controlling nearly all simulated outbreak scenarios. However, real-world experience suggests that masking alone has often been insufficient to fully control SARS-CoV-2 transmission. A sensitivity analysis using lower masking effectiveness would strengthen the robustness of the conclusions.

Response: We thank Reviewer 1 for this important discussion on our choice of parameter values in the intervention scenarios. In a sensitivity analysis, we tested a lower and upper bound for the efficacy of mask wearing on the close-contact transmission rate (Table 3). In the least favorable scenario, we explored a reduction of 17% of the close-contact transmission rate in mask wearers, along with a reduction of 80% of their exhalation rate of infectious aerosols. In Supplementary Figure 12, we show that changing the efficacy of masking does not change the ranking of the interventions for ICU1. In ICU2, the ranking is overall similar, except for two interventions among the middle-ranked interventions (targeted masking and targeted masking + ventilation within patient rooms) that are swapped in the most favorable and the least favorable scenarios compared to the main analysis. This sensitivity analysis confirms that universal masking, if correctly implemented, remains the best intervention even when considering lower mask efficacy, notably during close-proximity interactions.

5. In the simulations, transmission rates were varied to generate different scenarios. It would be helpful to clarify how these transmission rate settings relate to the basic reproduction number (R_0). Providing a mapping between the model parameters and implied transmissibility would help readers better interpret the simulated scenarios.

Response: We thank Reviewer 1 for this suggestion to use a metric that is more meaningful for the readers. Our modelling framework does not allow us to analytically derive a basic reproduction number, which will depend on the close-proximity contact network as well as on the chosen index case, whether a patient or a HCW. However, following Reviewer 1's suggestion, we used a simulation approach to translate transmission rates into values of basic reproduction numbers. More specifically, we simulated secondary transmission events along the close-proximity interactions network over the infectious period of the index case. We repeated these simulations 5,000 times while randomising over all possible index cases, meaning that the reproduction number is calculated on average between 50 and 90 times for each participant in ICU1

and ICU2 respectively. Then, we calculated the median reproduction number with its 95% prediction interval for each ICU. We now report reproduction numbers for patient and HCW index cases in Supplementary Table 3.

S3 Table. Resulting R_0 for each transmission scheme stratified by category of index case. For each network and transmission scheme, we simulated *n*=5,000 epidemics over the infectious period of the index case and reported the mean number of secondary cases infected by the index case with its 2.5 and 97.5 percentiles. We stratified the reproduction number by the category of the index case since the medical staff, paramedical staff, and patients have different contact patterns and roaming behavior in the ward, determining the frequency of transmission opportunities. For each simulation, the index case was randomly selected among the individuals present in the ICU the first day of the simulation.
Scheme 1: β_e = 1/45 new cases per infectious aerosol per day and β_c = 0.75 new case per contact per day; Scheme 2: β_e = 1/60 and β_c= 1.00; Scheme 3: β_e = 1/70 and β_c = 1.25; Scheme 4: β_e = 1/100 and β_c = 1.50; and Scheme 5: β_e = 1/150 and β_c = 1.75.

	**Transmission scheme**	**Medical staff**	**Paramedical staff**	**Patient**	**Global**
**ICU1**	Scheme 1	1.57 (1-4)	1.54 (1-4)	2.38 (1-5)	1.64 (1-4)
	Scheme 2	1.56 (1-3)	1.55 (1-4)	2.30 (1-5)	1.64 (1-4)
	Scheme 3	1.58 (1-4)	1.53 (1-4)	2.35 (1-5)	1.63 (1-4)
	Scheme 4	1.60 (1-4)	1.56 (1-4)	2.25 (1-5)	1.64 (1-4)
	Scheme 5	1.57 (1-4)	1.53 (1-4)	2.47 (1-5)	1.65 (1-4)
**ICU2**	Scheme 1	1.42 (1-3)	1.51 (1-4)	1.49 (1-3)	1.48 (1-3)
	Scheme 2	1.45 (1-4)	1.50 (1-3)	1.46 (1-3)	1.48 (1-3)
	Scheme 3	1.42 (1-4)	1.52 (1-4)	1.53 (1-3)	1.49 (1-4)
	Scheme 4	1.44 (1-4)	1.53 (1-4)	1.49 (1-3)	1.49 (1-4)
	Scheme 5	1.44 (1-4)	1.53 (1-4)	1.49 (1-3)	1.50 (1-3)

These R_0 values and Supplementary Table 3 are referred to in the Results section.

Result (Lines 186-192) : The relative contributions of the long- and short-range transmission routes to nosocomial respiratory epidemics remain poorly characterized and very likely depend on the pathogen. Here, we explore how the relative importance of one or the other transmission route modulates transmission dynamics and changes affected populations. We define five schemes, detailed in Table 1, that give more importance to one or the other transmission route, while maintaining similar global transmission intensity

within the ICU (Fig 3B and S5 Fig). R_0 ranges between 1.5 and 2.5 depending on the index case, the ICU, and the scheme (S3 Table).

6. In the generated contact networks, HCWs account for the majority of individuals. In real ICU settings, when HCWs are identified as infected, protocols typically remove them from patient care to reduce risk to patients. While such reactive interventions may be less relevant for pathogens with frequent asymptomatic carriage (e.g., antimicrobial-resistant bacteria), they are relevant for SARS-CoV-2. A discussion of how such policies might influence transmission dynamics in the modeled setting would be valuable.

Response: We thank Reviewer 1 for this interesting point of discussion. Indeed, the detection of a positive case among HCWs or patients often leads to the implementation of reactive interventions. Such interventions may rely on case isolation (patient isolation when the case is a patient, or sick leave for HCWs) and/or contact precautions that aim to reduce the transmission to other individuals (reinforced hand hygiene with glove wearing, gown wearing, reinforced mask wearing using FFP2 masks). Importantly, for respiratory viruses, infected HCWs do not necessarily take a sick leave, especially as it would lead to staffing issues in the ward. Besides, sick leaves do not necessarily coincide with the symptom onset or the start of the infectious period. Rather, there is a delay during which HCWs can transmit. As a consequence, illness presenteeism is widespread and generates a risk of nosocomial transmission for patients [3–5]. For example, in the SIREN cohort (prospective cohort of HCWs in the UK), about 65% of HCWs who tested positive for RSV, influenza virus and/or SARS-CoV-2 did not take a sick leave during winter 2023/24 [5]. Nonetheless, symptomatic HCWs who do not take a sick leave typically strengthen IPC measures such as hand hygiene and mask wearing.

All in all, these interventions do not only impact the level of transmission upon at-risk contacts, but also modify the contact network. For example, symptomatic HCWs would take a sick leave and be replaced by a temporary or permanent HCW during the time of the leave which would modify the network. Here, we made the simplifying assumption that the contact network is not rearranged when a patient or a HCW develops symptoms. Indeed, implementing such network rearrangements after symptom onset would not be trivial, and would require data to make correct hypotheses on behavioral changes. By not accounting for symptomatic HCW sick leaves, we might overestimate the transmission risk in the baseline scenario. On the other hand, the replacement of HCW on sick leave may increase the transmission risk by increasing the number of contacts between patients and HCWs if the HCW is replaced by a permanent staff and potentially if a temporary staff is hired as they were shown to comply less to hand hygiene for example [6]. We have extended the discussion section to integrate these elements.

Discussion (lines 339-350): We acknowledge other limitations in our modelling choices that presumably modulate transmission intensity and the efficacy of the tested interventions. First, outbreak dynamics in each transmission scheme and intervention scenario were derived from the introduction of a single index case in the ward. This might not be realistic as multiple introductions in hospital wards regularly occur during periods of seasonal epidemics of respiratory infections. This assumption was made to reduce the stochasticity and noise in our estimates. It presumably leads to an over-estimation of intervention efficacy, but this should not impact the ranking of the interventions. Second, we did not consider behavioral change in the close-proximity interaction network after the symptom onset of infected individuals. In real life situations, HCWs may pay more attention to hand hygiene, wear a mask or take sick leave, although illness

presenteeism is a common phenomenon (44–46). For symptomatic patients, they are generally isolated from other patients. By not accounting for such network rearrangement, we might have misestimated transmission levels.

7. The use of the term "long-range" transmission may not be entirely accurate in the context of the model. Aerosol transmission in the simulations appears to occur within the same room rather than over large spatial distances. Its main effect is to allow transmission among individuals who do not have direct spatiotemporal overlap in close contact. Clarifying the intended meaning of this term would improve precision.

Response: We thank Reviewer 1 for bringing up the potential confusion that could arise from the terminology used in the manuscript. We used the terminology from the 2021 review by Leung [7], a highly cited publication, and the follow-up comment by Leung and Milton published in 2024 [8]. Accordingly, long-range transmission merely refers to transmission events that occur when the infectee is at a distance of the infector, rather than transmission due to the long-distance travelled by the aerosol from its emission point. This terminology is well-adapted to our modelling strategy, as we model transmission during close-proximity contacts between infectors and susceptible individuals and exposure to air-suspended aerosols without tracking who exhaled these aerosols. However, we agree that we are not addressing the question of airborne circulation through airflows here. We have clarified this point in the abstract and the introduction so that it would be clear for all readers.

Abstract (lines 46-50): Nosocomial transmission of respiratory infections poses a major threat to patient safety, while also affecting healthcare workers' (HCW) health, generating substantial costs for hospitals. These infections spread through both close-proximity interactions at short distances, and via aerosols that remain suspended in the air, enabling long-range transmission within a room when a susceptible individual is at a distance from an infectious individual.

Introduction (lines 94-101): This process can be divided into two routes: the short-range route and the long-distance route [7–9]. The short-range route corresponds to transmission during close-proximity interactions between an infectious individual and a susceptible one. This includes transmission by the inhalation or deposition of aerosols, direct physical contacts and indirect contacts through fomites. Conversely, long-distance transmission occurs when a susceptible individual is at a distance of an infectious individual. It can result either from the exposure to pathogen-containing aerosols that remain suspended in the air, or through fomites previously deposited on environmental surfaces. The long-range route is characteristic of contaminated indoor spaces.

Results (lines 154-158) : Susceptible individuals get infected either during contacts with infectious individuals or following the inhalation of infectious aerosols in the rooms they visit. These two transmission routes feed into the global force of infection. Infectious aerosols within a room are assumed to be well-mixed and no airflow connects the rooms. The dynamics of their concentration within rooms is governed by an exponential decay due to pathogen inactivation and by a linear increase when infectious individuals are present in the room.

Reviewer 2

Thank you for the opportunity to review this paper.

This paper proposes a novel stochastic SEIR-type epidemiological model that integrates both short-range (close-contact) and long-range (aerosol) transmission routes of respiratory pathogens in hospital ICUs. The framework is applied to proximity sensor data from two French ICUs (Nods-Cov-2 study), using SARS-CoV-2 as a case study. The authors define five transmission "pathways" that vary the relative contribution of each route while maintaining similar overall epidemic intensity, and evaluate seven intervention scenarios including masking, hand hygiene, and ventilation.

The paper addresses an important gap: most nosocomial epidemiological models treat transmission as contact-driven, while biophysical and CFD models capture aerosol dynamics but over very short timescales and without population-level epidemic dynamics. Bridging these two traditions is valuable and timely, particularly in the post-COVID era. The framework of varying the relative contributions of short- vs. long-range routes and then evaluating tailored interventions is a practical and policy-relevant contribution. The location reconstruction algorithm derived from close-proximity sensor data is a useful methodological contribution for future studies.

However, I have several concerns regarding the clarity of the underlying hypothesis, the treatment of infected healthcare workers in the model, and the parameterisation of intervention scenarios which I detail below.

Response: We thank Reviewer 2 for their appreciation of the study.

Recommendation
I recommend **major revision**. The modelling framework is a valuable contribution, but the paper would benefit from: (1) a clearer articulation of the scientific question and the limitations of a scenario-based approach; (2) the inclusion of HCW isolation/removal as a realistic model feature; (3) a critical re-evaluation of the hand hygiene intervention parameterisation, which currently relies on community-setting evidence that is poorly suited to the model's SARS-CoV-2 ICU application; (4) presentation of intervention effectiveness stratified by HCWs, not only patients; and (5) an expanded discussion of the inter-ward implications of HCW infection via the aerosol route.

Major Comments
1. Clarify the underlying hypothesis and study framing
The specific scientific question this model is designed to answer is not clearly articulated. The paper appears to operate between several possible aims: (a) demonstrating that the relative contribution of short- vs. long-range transmission matters for choosing optimal interventions; (b) showing that long-range aerosol transmission plays a non-negligible role in nosocomial outbreaks; or (c) providing a framework that can identify which route dominates given appropriate data. The current framing conflates these, and the study design, which assumes different route contributions rather than inferring them from data, can only address (a). I would recommend that the authors explicitly state that this is a scenario-based simulation study and clarify what can and cannot be concluded from it. If the goal is ultimately to build an inferential framework, a discussion of what empirical data (e.g., genomic reconstruction, environmental sampling) would be needed to estimate the relative contributions of each route would strengthen the paper considerably.

Response: In light of this comment, we have made modifications throughout the manuscript to better highlight the main aim of the paper: developing a novel model that accounts for both transmission routes as well as contact data. The model is then used to evaluate intervention strategies by accounting for those mechanisms and identify flexible strategies that are effective in different behavioral and biological contexts. We notably modified the Abstract, the Author summary, and the Introduction.

Abstract (Lines 46-58): Nosocomial transmission of respiratory infections poses a major threat to patient safety, while also affecting healthcare workers' (HCW) health, generating substantial costs for hospitals. These infections spread through both close-proximity interactions at short distances, and via aerosols that remain suspended in the air, enabling long-range transmission within a room when a susceptible individual is at a distance from an infectious individual. The relative contribution of each transmission route is pathogen-dependent. However models distinguishing them remain scarce, limiting the design of effective intervention strategies. Here, we propose a novel agent-based stochastic model of respiratory pathogen transmission in a hospital ward that integrates both transmission routes together with contact patterns and individual movements. After informing our model with real close-proximity interaction data collected in two French intensive care units, we simulate a range of combinations of short- and long-range transmission levels to investigate their differences. Selecting parameter values that keep overall ward transmission intensity stable across combinations, the model is used to further evaluate the impact of intervention strategies on incidence risk.

Author summary (Lines 71-76): We introduce a novel epidemiological model of nosocomial outbreaks of respiratory infections that integrates short- and long-range transmission routes with close-proximity interactions and individual movements between ward rooms. Using simulations, we illustrate how the relative importance of the two transmission routes affects the dynamics of nosocomial transmission and what the implications are for the design of infection prevention interventions that should remain effective regardless of the dominant transmission route or the ward organization.

Introduction (Lines 125-148): Interestingly, two studies have tried to evaluate the relative contribution of the short-range, long-range, and fomite routes in hospital wards using data from nosocomial SARS and MERS-CoV outbreaks [10,11]. However, in these studies, many parameters were calibrated with values from the literature without exploring their impact in a sensitivity analysis, and the grid search approach used for parameter estimation does not guarantee an optimal exploration of the parameter space, limiting the interpretability of their results. A recent study integrated a CFD component into a transmission chain reconstruction model to evaluate the role of long-range transmission in SARS-CoV-2 nosocomial circulation [12]. They calibrated their model over more than two years of data but had no access to HCW information, and thereby overlooked a major actor of nosocomial transmission [13]. Since the relative contributions of the two routes remains little documented in healthcare settings, there is a need for models coupling both transmission routes applied to nosocomial transmission, notably for intervention design.

From an epidemiological and public health perspective, an explicit description of the respective roles of the short- and long-range routes in nosocomial respiratory outbreaks is essential to develop effective control strategies [9]. Here, we propose a novel dynamic epidemiological model that describes the transmission of a range of respiratory pathogens in a hospital ward. After presenting the model that accounts for complex

temporal inter-individual contact networks, individual movements between ward rooms, and exposure to pathogen-laden aerosols suspended in the indoor air, we apply it to records of close-proximity interactions in two French intensive care units (ICUs). Through simulations, we explore how varying the levels of the short- and long-range transmission routes actually impacts epidemic dynamics, using SARS-CoV-2 as a case study. Based on such dynamics, the effectiveness of IPC interventions is evaluated, including hand hygiene, mask wearing, and air ventilation, to identify the best strategy for each contribution level of the two transmission routes.

Response: As pointed out by Reviewer 2, we think that our model could be adapted in an inference framework to quantify transmission rates for each route, providing there is both contact and infection data available. We added a paragraph in the Discussion explaining the empirical data needed and possible research perspectives.

Discussion (Lines 313-319): Here, we propose a novel epidemiological model that describes short- and long-range transmission routes, and incorporates the highly-structured contact networks in hospital settings. We think that the restricted parameter space of our model makes it well-suited to the future development of an inference framework and quantifying transmission rates associated with each route compared to CFD-based models [12,14]. Longitudinal data on close-proximity interactions, infections in patients and HCWs, and environmental contamination of the indoor air of ward rooms would be necessary to inform the model and provide reliable estimates.

2. Identifiability and the long-range aerosol route

The model's long-range transmission route captures exposure to pathogen-laden aerosols accumulated in room air. However, the well-mixed box model per room cannot distinguish aerosol inhalation from other environmental contamination pathways (e.g., fomite transmission via contaminated surfaces). This is relevant because the interventions that address these mechanisms differ (ventilation vs. surface disinfection). A discussion of identifiability, specifically whether and how the aerosol route can be distinguished from environmental contamination given available data, would be valuable, particularly if this framework is to be used for intervention planning for other pathogens.

Response: We agree with Reviewer 2 that long-range transmission through fomites can be of major importance for some pathogens. Being able to quantify the importance of transmission through fomites compared with aerosols is valuable to evaluate and design control strategies. The current formulation of the model assumes that the infection risk increases linearly with the quantity of aerosols contained in the volume of air inhaled by susceptible individuals: $\lambda_e^j(t) = \beta_e \sum_{k \in R^j} 1_{\{r^j(t)=k\}} B \frac{E^k(t)}{V^k}$. This formulation can indeed be interpreted in two ways: 1) exhaled aerosols are responsible for exposure through inhalation only and there is no transmission through fomites, or 2) the same mechanism underlies transmission through fomites and through suspended aerosols. Thus, our model formulation does not allow distinguishing transmission through fomites which would require a specific formulation for fomite generation, stability, and exposure. We have changed the limitations paragraph of the Discussion section on the generalizability

of our model and results to address this point. It now touches upon the lack of identifiability between environmental and aerosol routes in our model.

Discussion (lines 374-377): We also did not explicitly model transmission through fomites here but rather combined in a single term the inhalation of suspended aerosols and surface contamination. To differentiate these two acquisition routes, a specific exposure function for each could be formulated in the future, with possible parameterisation from the literature or data from biological sampling of surfaces.

3. Treatment of infected HCWs: absence of isolation or workforce depletion

A significant concern is the apparent absence of any mechanism for removing infected HCWs from the workforce. The model appears to allow infectious HCWs to continue working, making contacts, and shedding aerosols throughout their infectious period. This has several consequences:

First, it inflates HCW-driven transmission. The reported secondary attack rates of 50 to 76% among HCWs likely reflect this unrealistic continued exposure. In practice, symptomatic HCWs would be identified and removed from the care environment, truncating transmission chains. Second, there is an internal inconsistency: the paper tests universal masking "from symptom onset of the index case," implying awareness of who is infectious. If the model can identify symptom onset, it should also be able to implement HCW isolation or sick leave at that point. Third, the finding that the long-distance aerosol route disproportionately affects HCWs is one of the paper's most interesting results, but its consequences are not fully explored. Infected HCWs removed from the workforce would reduce staffing ratios and potentially compromise patient care, a workforce depletion effect that could feed back into epidemic dynamics. Implementing HCW removal upon infection (with or without a detection delay) would be a relatively straightforward model extension and would allow the authors to explore system resilience as an outcome.

Response: We agree with Reviewer 2 that not accounting for sick leaves in infected HCWs potentially inflates HCW-driven transmission. Nevertheless, a study focusing on strategies to replace HCWs in contexts of nurse shortage interestingly showed that reassigning patients to other working nurses would increase the risk of nosocomial transmission [15]. Besides, illness presenteeism is widespread, as has already been documented for common respiratory viruses during seasonal epidemics, although the studies did not quantify changes in infection prevention and control measures if a HCW is symptomatic [3–5]. This phenomenon is magnified for bacterial infections as we would expect HCWs to be asymptomatic carriers of the respiratory pathogen. We have changed the Discussion that now details this limitation.

Discussion (lines 339-350): We acknowledge other limitations in our modelling choices that presumably modulate transmission intensity and the efficacy of the tested interventions. First, outbreak dynamics in each transmission scheme and intervention scenario were derived from the introduction of a single index case in the ward. This might not be realistic as multiple introductions in hospital wards regularly occur during periods of seasonal epidemics of respiratory infections. This assumption was made to reduce the stochasticity and noise in our estimates. It presumably leads to an over-estimation of intervention efficacy, but this should not impact the ranking of the interventions. Second, we did not consider behavioral change in the close-proximity interaction network after the symptom onset of infected individuals. In real life situations, HCWs may pay more attention to hand hygiene, wear a mask or take a sick leave, although illness presenteeism is a common phenomenon [3–5]. For symptomatic patients, they are generally isolated

from other patients. By not accounting for such network rearrangement, we might have misestimated transmission levels.

4. Outcome metrics and intervention evaluation

The authors evaluate intervention effectiveness using cumulative incidence among patients (Figure 5A). However, a key finding of this paper is that the long-distance aerosol route disproportionately affects HCWs. If this is the case, then evaluating ventilation effectiveness primarily through patient outcomes will underestimate its value. Supplementary Figure 7 presents results stratified by individual category, which partially addresses this concern. However, this stratification should be presented in the main text rather than relegated to the supplement, given its centrality to the paper's narrative. Ideally, a composite metric that captures both direct health outcomes and system resilience (e.g., staffing capacity) would provide a more policy-relevant assessment of intervention value.

Response: Following Reviewer 2 comment, we have modified Figure 5A to integrate not only results on patients, but also on HCWs. We already detailed this result at lines 236-244 "In the case of ventilation, targeting rooms that are not accessible to patients is more effective in terms of patient incidence than targeting patient rooms (Fig 5A). Indeed, targeting rooms that are not accessible to patients reduces infections in HCWs, that is to say the first source of infection for patients in our analysis. When combined with targeted masking, ventilation is as effective as universal masking in terms of patient incidence and of HCW incidence as well (Fig 5A), but only when ventilation targets break rooms. Overall, coupling targeted masking with ventilation in break rooms reduces acquisition among HCWs as well as transmission from HCWs to patients (S9 Fig). This mixed intervention is thereby almost as effective as universal masking, although its efficacy tends to decrease in HCWs for schemes where the short distance route is predominant (Scheme 5; Fig 5A).", but we have now modified the Abstract and the Author Summary so that it appears among our key findings.

Abstract (Lines 58-66): We find that the predominance of one route over another has little effect on overall outbreak dynamics, though the impact on individuals varies markedly. Patients are mostly at risk of short-range transmission from HCWs, while HCWs are mostly affected by whichever route is predominant. This directly influences intervention effectiveness. Universal masking emerges as the most effective strategy, reducing both transmission routes. Its stringency can be relaxed with limited loss of effectiveness when combined with ventilation in relevant rooms. Importantly, interventions targeting HCWs, notably ventilation in rooms not accessible to patients, indirectly reduces incidence in patients, with a stronger effect when coupled with relaxed masking interventions. Finally, intervention ranking remains robust across parameter values, as confirmed by a sensitivity analysis.

Author summary (Lines 71-79): We introduce a novel epidemiological model of nosocomial outbreaks of respiratory infections that integrates short- and long-range transmission routes with close-proximity interactions and individual movements between ward rooms. Using simulations, we illustrate how the relative importance of the two transmission routes affects the dynamics of nosocomial transmission and what the implications are for the design of infection prevention interventions that should remain effective regardless of the dominant transmission route or the ward organization. We showed that HCWs are more affected than patients, constituting a major source of infection. Breaking transmission between HCWs can effectively reduce the infection risk in patients for example by combining mask wearing during interactions

with patients with ventilation in break rooms. Such strategies can offer an effective alternative to universal masking.

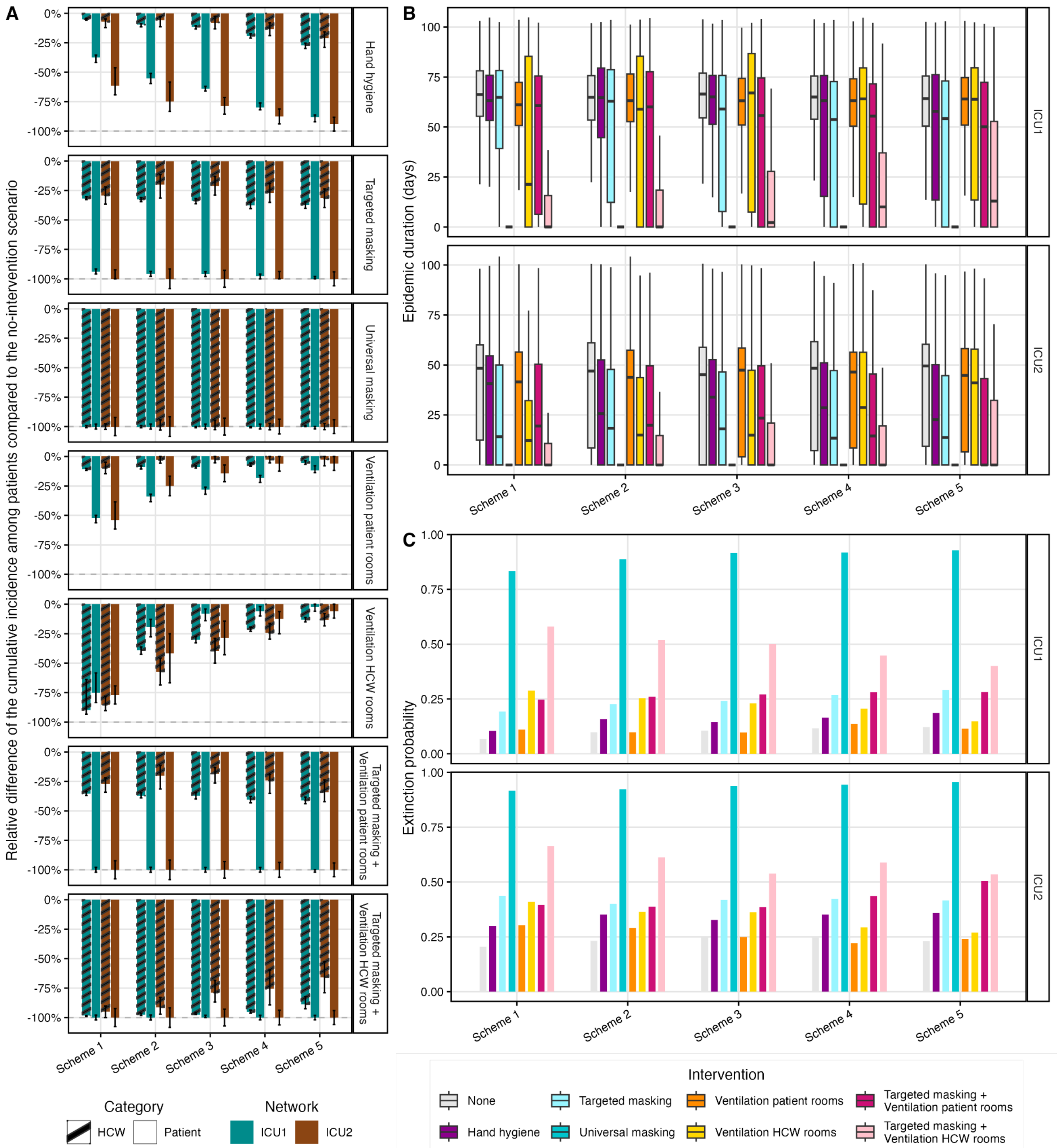

Fig 5. Impacts of control interventions on secondary attack rates, epidemic duration, and extinction probability across transmission schemes. (**A**) Relative difference of the 90-day cumulative incidence among patients and HCWs between the intervention scenario and the baseline scenario (with no intervention). In the hand hygiene scenario, the transmission rate between HCWs and patients was reduced by 14%. In scenarios with targeting masking, transmission rates among patients and between HCWs and patients was reduced by 70% and aerosol exhalation by 94%. In the universal masking scenario, transmission was reduced by 70% in all pairs of individuals, and aerosol exhalation by 94%. In scenarios

with ventilation, we increased ACH to 8. Negative values indicate a reduction in the cumulative incidence when an intervention is in place. Relative difference estimates and error bars are derived from the pseudo-median and the 95% CI of Wilcoxon tests, respectively. Reductions stronger than 100% are explained by the definition of the metric: the median of the cumulative incidence in the baseline scenario used as the reference can be higher than the 95% CI bounds of the difference. (**B**) Distribution of epidemic durations in the baseline and intervention scenarios. (**C**) Extinction probability in the baseline and intervention scenarios.
Scheme 1: β_e = 1/45 new cases per infectious aerosol per day and β_c = 0.75 new case per contact per day; Scheme 2: β_e = 1/60 and β_c= 1.00; Scheme 3: β_e = 1/70 and β_c = 1.25; Scheme 4: β_e = 1/100 and β_c = 1.50; and Scheme 5: β_e = 1/150 and β_c = 1.75.

5. HCWs as inter-ward bridges: expand in discussion
The model is confined to a single ICU, but one of its most striking findings, that long-range aerosol transmission disproportionately infects HCWs, has implications that extend beyond the ward. HCWs are the most mobile population in hospitals: they rotate between wards, float to other units, visit break rooms, cafeterias, and meeting spaces. If the long-range route is what primarily infects HCWs, then infected HCWs become the bridge for inter-ward and inter-department dissemination. This connects naturally to a metapopulation perspective on hospitals as networks of wards linked by HCW movement. I would encourage the authors to expand on this in the discussion, as it represents both a limitation of the current single-ward model and a compelling direction for future work, and it follows directly from their own results.

Response: We thank Reviewer 2 for this interesting comment. HCWs infections are often poorly analysed because it is difficult to collect data on their infections, contacts and movements. For example, the role of patient transfers between wards of the same hospital on nosocomial transmission has already been documented but less has been done on HCWs due to the difficulty to collect precise data on their schedules and an extensive list of their close-proximity contacts [16]. Indeed, HCWs are in contact with HCWs and patients from other wards, their family members, and the general community when they visit public spaces, but many of these contacts are missed in studies using wearable sensors. Interestingly, the characteristics of the contacts made by HCWs outside of their ward may vary by type of HCW as suggested by a survey carried out among HCWs of three hospitals in Canada that collected the rooms HCWs visited and their contacts [17]. All in all, hospital-level organization very probably affects intervention efficacy. In a recent study, the authors fitted a stochastic agent-based model to hospital data and estimated that between-ward mixing has the highest attributable fraction for 13 out of 20 nosocomial SARS-CoV-2 infections [18]. This result highlights that between-ward transmission may be a key source of nosocomial infection. We have extended our Discussion to integrate these elements.

Discussion (Lines 350-353): Third, the Nods-CoV-2 study covered ward-level interactions, although HCWs may have contacts in other wards. It has been recently shown that between-ward mixing was the most probable source of nosocomial SARS-CoV-2 infections in a large UK hospital [19]. New data covering multiple wards are necessary to account for this transmission mechanism.

6. Hand hygiene intervention: questionable parameterisation, relevance for SARS-CoV-2, and supporting citations

The parameterisation of the hand hygiene intervention raises several concerns. The authors model hand hygiene as a 17% reduction in the contact transmission probability from HCW to patient ($\beta c, HCW \rightarrow patient$), citing Aiello et al. (2008), Jefferson et al. (2008), and Ross et al. (2023).

Setting mismatch: All three references study hand hygiene in community settings: households, schools, daycare centres, and low- and middle-income countries. None examine HCW hand hygiene in ICUs or hospitals. The transmission dynamics, baseline hygiene practices, pathogen exposure intensity, and contact patterns differ fundamentally between these settings.

Outcome mismatch: The cited studies use broad syndromic outcomes ("respiratory illness," "ILI") that bundle together many pathogens with different transmission routes (rhinoviruses, adenoviruses, RSV, influenza). The 17 to 21% reductions reported likely reflect a composite of reduced fomite transmission, self-inoculation, and behavioural co-interventions. Applying this estimate to a single mechanistic parameter (βc) implies the entire effect operates through direct contact transmission, which is almost certainly not the case.

Pathogen mismatch: The model is parameterised for SARS-CoV-2, for which there is now strong evidence that airborne transmission is the dominant route and fomite transmission plays a negligible role (Greenhalgh et al., *The Lancet* 2021; Meyerowitz et al., *Annals of Internal Medicine* 2021; Wee et al., *F1000Research* 2021). Hand hygiene is primarily effective against fomite-mediated transmission. The inclusion of hand hygiene as an intervention for SARS-CoV-2 contact transmission requires stronger justification. If the authors intend the model to be general to respiratory pathogens, they should acknowledge that the hand hygiene effect estimate is likely an overestimate for SARS-CoV-2 specifically. While the authors performed sensitivity analyses, the realistic focus should be on lower effectiveness rather than higher ones.

Supporting citation. Relatedly, the authors cite Evans et al. (2022) to support the claim that transient hand carriage in HCWs might be an important route for viruses. However, Evans et al. models an "indirect patient-to-patient" pathway that encompasses multiple mechanisms (HCWs as vectors, contaminated fomites) without isolating hand carriage as the specific driver. The evidence for hand-mediated transmission of respiratory viruses is considerably weaker than for bacterial pathogens (e.g., MRSA, VRE, *C. difficile*) or enteric viruses (e.g., norovirus), where hand contamination is well-documented and central to transmission. The authors should either cite more specific evidence for hand carriage of respiratory viruses or reframe this limitation.

Response: Reviewer 2 is correct that we did not clarify the lesser level of evidence of hand hygiene on SARS-CoV-2 transmission specifically. We now refer to a meta-analysis of risk factors of SARS-CoV-2 infection among HCWs where the authors quantified an OR of 1.17 (95% CI: 0.79-1.73) when hand hygiene is inadequate [20]. This OR is not significant which, according to the authors, could come from the heterogeneity in hand hygiene reporting in the included studies. Accordingly, we now modulate our justification of testing hand hygiene for SARS-CoV-2 in the Methods and changed the references in Table 2. We changed parameter values explored in the main and sensitivity analysis to match the OR from Bansal et al. (2025) [20]. We now use a reduction of 14% (=1-1/1.17) in the main analysis and a maximal reduction of 42% (=1-1/1.73). For the lowest reductions, we decided to keep 5% as a reduction of 0% would not impact transmission dynamics. We now justify this choice in the methods. We also changed the intervention scenario so that transmission is coherently reduced in both directions (HCWs to patients and patients to HCWs).

Methods (Lines 521-532): We define and test seven strategies: (*i*) improved hand hygiene of HCWs, which limits the short-range transmission risk between HCWs and patients, (*ii*) HCWs wear masks when they are in patient rooms or in interaction with patients and patients wear masks when they are symptomatic, which reduces infectious aerosol exhalation in patient rooms and the corridor and the risk of short-range transmission during patient-patient and patient-HCW interactions, (*iii*) universal mask wearing after the symptom onset of the index case, (*iv*) improved ventilation in rooms that can be visited by patients (patient rooms and corridor), (*v*) improved ventilation in rooms that are not accessible to patients (nursing station, medical office, medical and paramedical staff rooms), (*vi*) a mixed intervention that combines interventions (*ii*) and (i*v*), and (*vii*) a mixed intervention that combined (*ii*) and (*v*) (Table 2). Although there is non-significant evidence of hand hygiene protective effect against SARS-CoV-2 nosocomial transmission, we included this strategy as it is rarely well implemented and is part of the common infection prevention and control strategies [20].

Response: We also reformulated our statement on the role of transient hand carriage in nosocomial transmission of viral pathogens in the Discussion.

Discussion (Lines 353-356): Fourth, we did not integrate the transient hand carriage in HCWs in our model, although this is a potential route for viruses [13], and has been shown as a major route for bacteria [21]. Accounting for this route would increase the impact of hand hygiene and material disinfection on transmission dynamics in our simulations.

Minor Comments

7. Data availability and reproducibility

I understand that the close-proximity sensor data from the Nods-Cov-2 study cannot be made publicly available. However, to support reproducibility and allow other researchers to test or extend this framework, the authors should at minimum provide mock or synthetic data that preserves the structure and format of the input data without disclosing individual-level information.

Response: We thank the reviewer for pointing out this important consideration. We have added synthetic data that have the same structure and format as the real data from Nods-Cov-2 and now refer to them in the data availability statement.

Data availability statement: Interaction data from the Nods-Cov-2 project are not publicly available but can be made available from the Institut Pasteur (contact point: Prof Dider Guillemot) on reasonable request. Instead, we provide synthetic contact networks, and the corresponding staff and patient presence information that are available at https://github.com/MESuRS-Lab/aerosol-epi-model.

8. Ventilation intervention contextualisation

The scenario of increasing ventilation to 8 ACH in patient-accessible rooms would benefit from contextualisation: what are typical baseline ACH values in French ICUs? What is the feasibility and cost of achieving 8 ACH? Is this consistent with current guidelines (e.g., WHO or French national standards)?

Response: We thank the reviewer for this interesting question. To the best of our knowledge, there is no information in the literature about the average ACH values in French ICUs. Nevertheless, the French

Society for Hospital Hygiene (SF2H) recommends an ACH of 6 in hospitals that should exceed 10 in specific settings (rooms with immunocompromised patients, rooms with patients with airborne infection, ICU wards, waiting rooms in emergency departments etc…). According to a report by the General Inspectorate of Social Affairs, the ageing rate of hospital buildings in France reached 50% in 2024 and it is expected to accelerate due to the limited investments in energy-efficient building refurbishment. This suggests that a large part of hospitals may not meet recommendations from the SF2H. An ACH of 8 lies in-between the baseline recommendation for hospitals and strict airflow recommendations in specialised rooms by the SF2H.

9. Generalisability beyond SARS-CoV-2

The authors state that the model is applicable to other airborne pathogens (RSV, influenza), but the parameterisation is entirely SARS-CoV-2-specific. The relative importance of aerosol vs. contact routes, latent periods, infectious periods, and aerosol stability vary substantially across pathogens. One advantage of this framework is its potential generalisability; however, demonstrating this, either with an additional pathogen parameterisation or by more carefully delineating which conclusions are SARS-CoV-2-specific vs. general, would strengthen the paper.

Response: As stated by Reviewer 2, the modelling framework can be easily extended to other respiratory infections. Indeed, the parameters that are specifically informed to match SARS-CoV-2 epidemiology are: the incubation period, the infectious period, the existence of a pre-symptomatic period, the exhalation rate, and the aerostability of the pathogen. In parallel, we explored the impact of the importance of long- and short-range transmission routes in the main analysis using combinations of values that could apply to a variety of pathogens. In the sensitivity analysis, we also evaluated the role of pathogen aerostability, again exploring a wide range of possible values. This sensitivity analysis suggests that the general conclusions of our simulations could be generalisable to other respiratory viruses like influenza viruses and the respiratory syncytial virus. To further showcase our point, we now provide analyses using influenza viruses as a case study. We chose influenza virus-specific parameter values and simulated nosocomial outbreaks with and without interventions. The effects of the interventions are qualitatively similar. We mostly observe a lower effectiveness of control interventions in specific conditions in ICU2 due to the low transmission of the virus.

Supporting information:

4. Influenza virus case study

To further demonstrate the flexibility of our model and the generalizability of our results, we simulated nosocomial outbreaks using parameter values characteristic of influenza viruses (S4 Table). Given that the importance of the close-proximity route compared with the long-distance route has been little quantified for influenza viruses, we explored transmission dynamics for the values of the close-proximity transmission rate and the long-distance acquisition rate chosen in the grid search approach for SARS-CoV-2. We informed the other parameters, namely the exhalation rate, the half-life of aerosolized influenza viruses, the incubation period, and the infectious period, from the literature. Importantly, influenza A viruses have been more studied than influenza B viruses, we preferentially chose parameter values of influenza A viruses.

Presymptomatic transmission has been shown to play a role in influenza A virus transmission [22,23]. In their modelling study, Zhang et al. [23] estimated that presymptomatic transmission occurred on average

0.7 days before symptoms onset in 16% of flu cases when using the incubation period from the meta-analysis by Lessler and colleagues [24]. Thus, we implemented pre-symptomatic transmission similarly to the main analysis on SARS-CoV-2. Presymptomatic transmission occurred in 16% of the cases and could occur at most 0.7 days before symptom onset.

S4 Table. Parameter values used to parameterize the epidemic simulation model for influenza viruses. The simulation model is parametrized using values taken from the literature, except for β_c and β_e that we calibrate using a grid search approach. Importantly, we inform the inactivation rate of aerosols using the estimates of half-life for seasonal influenza A viruses from Rockey et al. (2024) that lie around 0.55 hours. If we consider that the air of a room is completely renewed when the quantity of aerosols has decreased by more than 99%, then a half-life of 0.55 hours is equivalent to an air change per hour (ACH) of 0.28.
Scheme 1: β_e = 1/45 new cases per infectious aerosol per day and β_c = 0.75 new case per contact per day; Scheme 2: β_e = 1/60 and β_c= 1.00; Scheme 3: β_e = 1/70 and β_c = 1.25; Scheme 4: β_e = 1/100 and β_c = 1.50; and Scheme 5: β_e = 1/150 and β_c = 1.75.

Parameter	Description	Value					Unit	Reference
		Scheme 1	Scheme 2	Scheme 3	Scheme 4	Scheme 5		
β_c	Close-proximity transmission rate	0.75	1.00	1.25	1.50	1.75	No. of new cases per contact per day	Grid search
β_e	Long-distance acquisition rate	1/45	1/60	1/70	1/100	1/150	No. of new cases per aerosol per day	Grid search
ν	Exhalation rate of infectious aerosols	2.5e4					RNA copies per 30 min	Yan et al., 2018 [25]
μ	Pathogen-laden aerosol removal due to pathogen inactivation	1.3 (0.55)					Rate per hour (half life in hour)	Rockey et al., 2024 [26]
$\Delta T_{incubation}$	Incubation period	Lognormal(log-mean=0.7, log-sd=0.2)					day	Lessler et al., 2009 [24]
$\Delta T_{infectious}$	Infectious period	U(1,3)					day	Zhang et al., 2026 [23]

In S14 and S15 Figs, we present the effects of control interventions on outbreak dynamics of an influenza-like virus. The results are comparable to the SARS-CoV-2 case study with universal masking outperforming the other interventions and targeted masking combined with ventilation in break rooms ranking second. For some conditions, we observe a limited reduction of incidence in ICU2 that comes from the low transmission

levels of the virus in the population, especially in patients and medical staff. Overall, this analysis shows that due to the close natural history between SARS-CoV-2 and influenza viruses, interventions perform similarly across transmission schemes. These results are presumably generalizable to other common respiratory viruses.

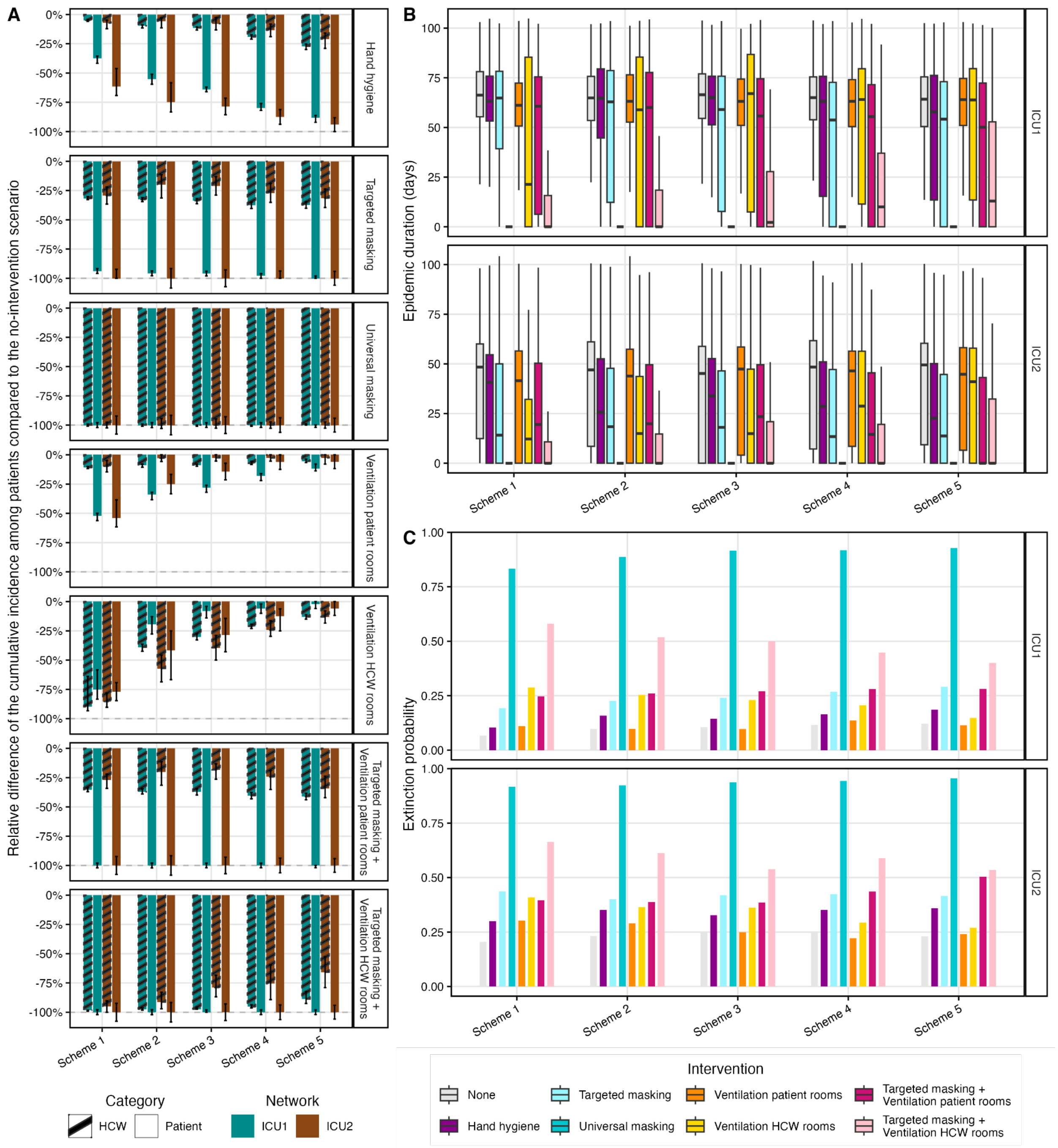

S14 Fig. Impacts of control interventions on secondary attack rates, epidemic duration, and extinction probability across transmission schemes for influenza viruses. (**A**) Relative difference of the 90-day cumulative incidence among patients and HCWs between the intervention scenario and the baseline scenario (with no intervention). In the hand hygiene scenario, the transmission rate between HCWs and patients was reduced by 14%. In scenarios with targeting masking, transmission rates among patients and

between HCWs and patients was reduced by 70% and aerosol exhalation by 94%. In the universal masking scenario, transmission was reduced by 70% in all pairs of individuals, and aerosol exhalation by 94%. In scenarios with ventilation, we increased ACH to 8. Negative values indicate a reduction in the cumulative incidence when an intervention is in place. Relative difference estimates and error bars are derived from the pseudo-median and the 95% CI of Wilcoxon tests, respectively. Reductions stronger than 100% are explained by the definition of the metric: the median of the cumulative incidence in the baseline scenario used as the reference can be higher than the 95% CI bounds of the difference. Importantly, the median cumulative incidence in patients in ICU2 for Scheme 2 is null when there is no intervention, thus we cannot compute relative reductions in this specific case. Nevertheless, absolute reductions are significantly different from 0 with a p-value < 0.001 for all interventions. (**B**) Distribution of epidemic durations in the baseline and intervention scenarios. (**C**) Extinction probability in the baseline and intervention scenarios.
Scheme 1: $\underline{\beta_e}$ = 1/45 new cases per infectious aerosol per day and $\underline{\beta_c}$ = 0.75 new case per contact per day; Scheme 2: $\underline{\beta_e}$ = 1/60 and $\underline{\beta_c}$= 1.00; Scheme 3: $\underline{\beta_e}$ = 1/70 and $\underline{\beta_c}$ = 1.25; Scheme 4: $\underline{\beta_e}$ = 1/100 and $\underline{\beta_c}$ = 1.50; and Scheme 5: $\underline{\beta_e}$ = 1/150 and $\underline{\beta_c}$ = 1.75.

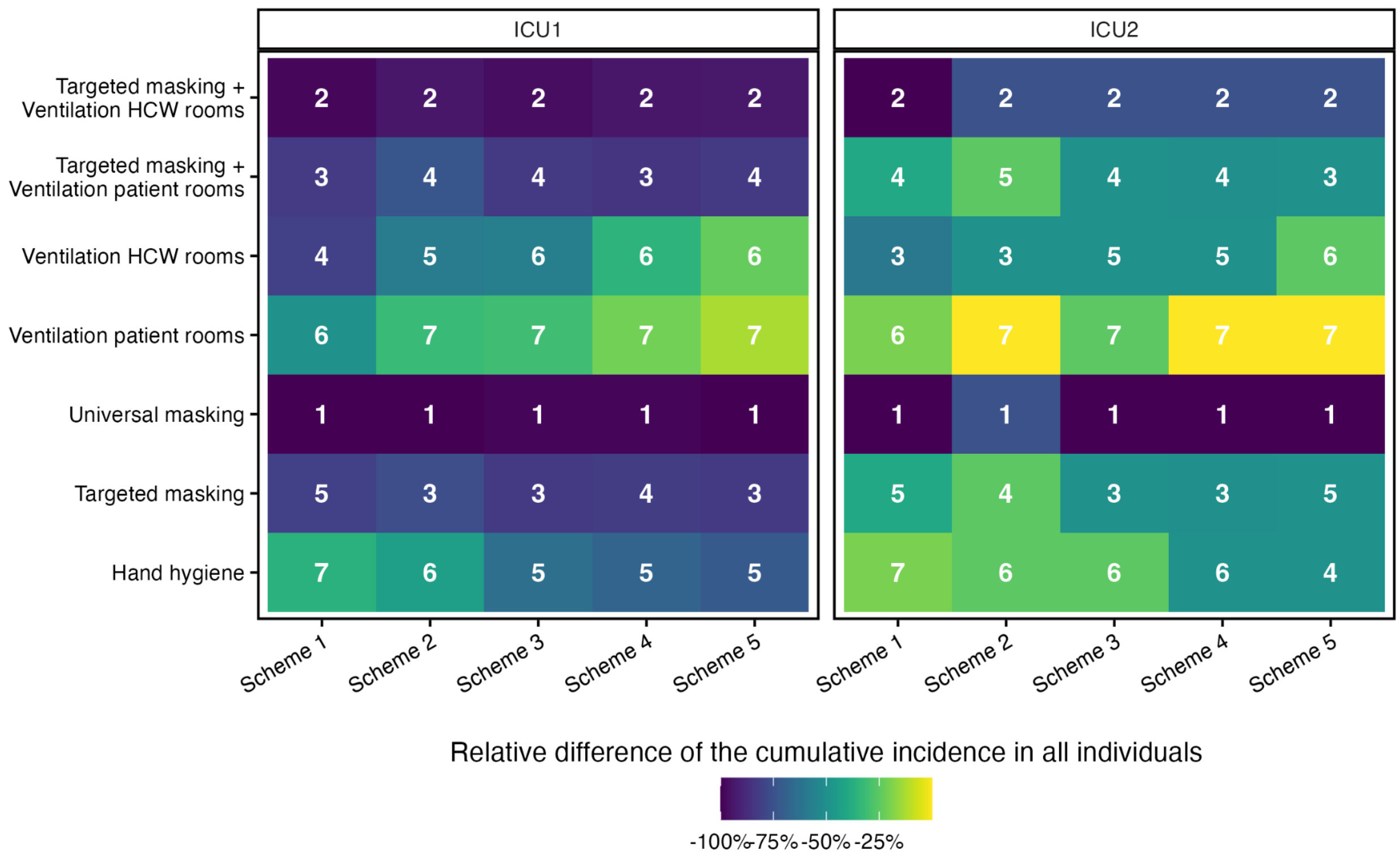

S15 Fig. Ranking of the most effective intervention against influenza virus transmission in reducing the overall cumulative incidence over 90 days. For each combination of ICU and transmission scheme, we rank the effectiveness of interventions based on the relative difference in the cumulative incidence over 90 days compared to the scenario without intervention.
Scheme 1: $\underline{\beta_e}$ = 1/45 new cases per infectious aerosol per day and $\underline{\beta_c}$ = 0.75 new case per contact per day; Scheme 2: $\underline{\beta_e}$ = 1/60 and $\underline{\beta_c}$= 1.00; Scheme 3: $\underline{\beta_e}$ = 1/70 and $\underline{\beta_c}$ = 1.25; Scheme 4: $\underline{\beta_e}$ = 1/100 and $\underline{\beta_c}$ = 1.50; and Scheme 5: $\underline{\beta_e}$ = 1/150 and $\underline{\beta_c}$ = 1.75.

Response: We now refer to these results at the end of the Results section. We better highlight the generalizability of these results in the Discussion section. Finally, we rapidly present the influenza case study in the Methods section.

Results (Lines 247-256): Biological characteristics of respiratory pathogens and intervention effectiveness against infection are difficult to quantify precisely, both in controlled experiments and in observational studies. The parameter values that we used in the main analysis (Table 1) do not reflect the full range of possible values for each parameter. To assess the impact of this uncertainty, we evaluate changes in nosocomial dynamics when using upper and lower bounds of pathogen aerostability, pathogen exhalation, and intervention effectiveness (Table 3). As expected, higher aerostability or higher exhalation rate of infectious aerosols increase the global SAR. Concerning interventions, increasing or decreasing their efficacy does not modulate overall dynamics (S10 Fig) and little affects the ranking of the most effective interventions (S11-S13 Figs). Finally, when we use influenza virus-like parameter values (S4 Table), the effects of the interventions are qualitatively similar (S14 and S15 Figs).

Discussion (Lines 265-272): Using a grid search approach, we have selected five transmission schemes giving more weight to one transmission route or the other while maintaining similar circulation intensity in the ward. Although we used SARS-CoV-2 for illustration purposes, the model can easily be extended to other airborne pathogens, as exemplified by our sensitivity analysis on influenza viruses. Our general conclusions are likely to be applicable to other respiratory viruses, such as the respiratory syncytial virus or influenza viruses, that have similar transmission characteristics as SARS-CoV-2 [24,27]. This is comforted by the sensitivity analysis on the aerostability of the pathogen showing little changes in the global SAR, as well as the evaluation of intervention strategies using influenza virus as a case study.

Methods (Lines 545-548): Finally, we apply our model to an influenza-like virus by parameterizing the model using values from the literature (S4 Table), mostly derived from influenza A viruses. We evaluate the effectiveness of interventions in both ICUs for patients and HCWs (S14 Fig), and rank them based on the reduction in cumulative incidence (S15 Fig). More details are available in the Supporting Information.

Editorial Comments

10. Terminology: “pathways” vs. “scenarios”

The term “pathway” to describe different parameter combinations giving varying weight to the two transmission routes is potentially confusing, as “pathway” in epidemiology often refers to a mechanistic route of transmission. Since these represent hypothetical parameter configurations chosen via grid search, the term “scenario” would be more appropriate and less ambiguous.

Response: We have changed the terminology throughout the manuscript as suggested by Reviewer 2, but we opted for the term “transmission scheme” so that the term “scenario” remains devoted to the intervention scenarios.

11. Figure-specific comments

Figure 2A: Please expand the caption to explain what the dots represent. The figure is somewhat interpretable but would benefit from a more detailed description of how individual movements are visualised.

Response: In the original version of the figure, dots represented when the individual entered/got out of a room and vertical lines represented the time units at which an individual was present in a room. When an individual stayed over a long time period, this resulted in a horizontal thick and colored band. To improve the clarity of the figure, we have modified the figure so that the location of the individual is simply represented by colored marks. We have also modified the figure caption.

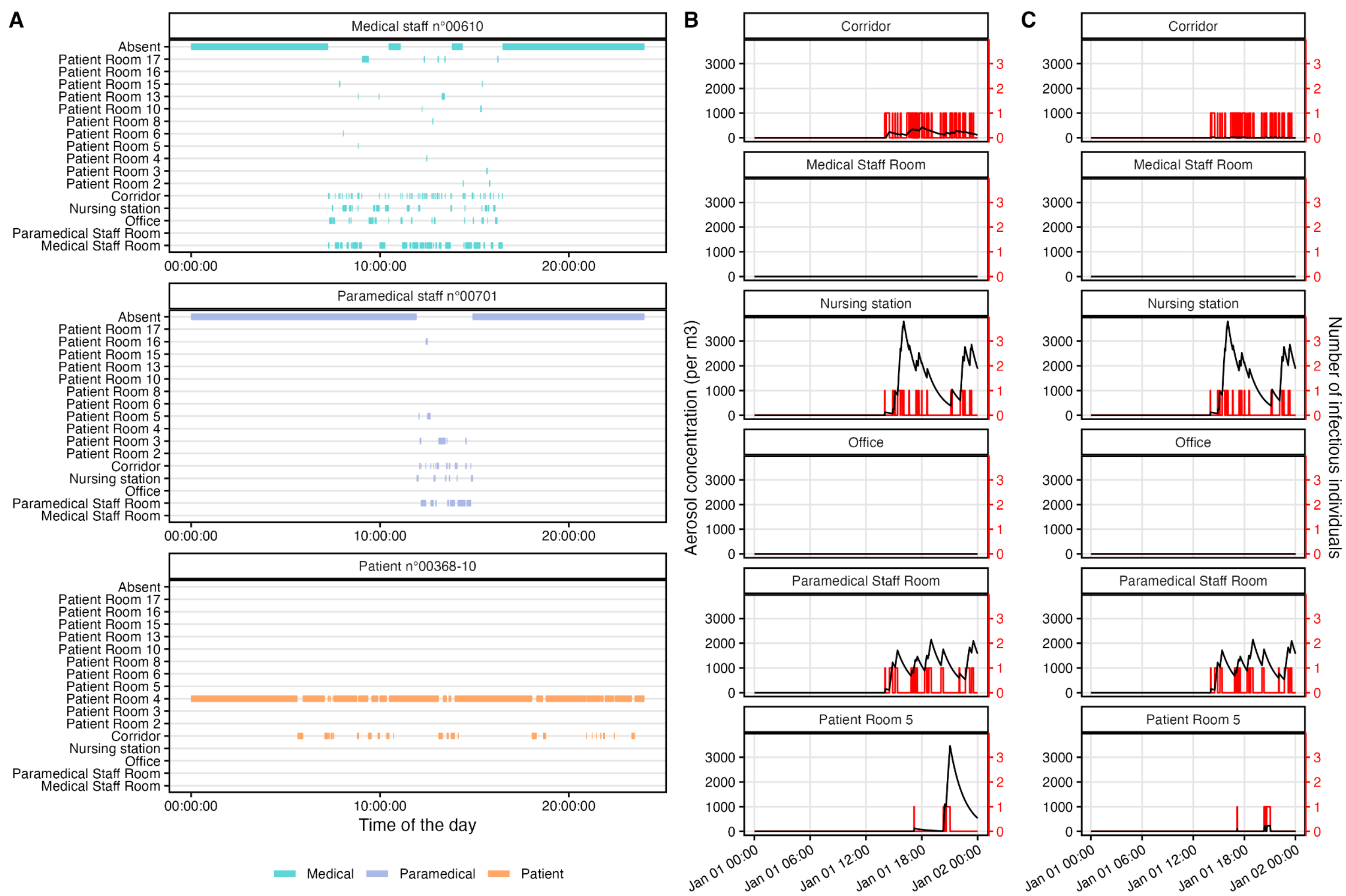

Fig 2. Illustration of simulation outputs. (**A**) Example of movements over one day for three randomly selected individuals among the medical staff, paramedical staff, and patients in ICU1. Colored marks indicate the location of the individual at each time step of the day. Thin marks indicate that the individual stayed over a short period of time, while long horizontal bands indicate a prolonged stay. (**B-C**) Example of simulated aerosol concentrations (in black) in a subset of rooms in ICU1 without intervention (**B**) and with improved ventilation in rooms accessible to patients (**C**). The number of infectious individuals present in the room is depicted in red at each time step. Aerosol concentration increases when infectious individuals are present and decreases otherwise due to pathogen inactivation. When increasing the ventilation to 8 air changes per hour (ACH) in rooms accessible to patients (corridor and patient room n°5), the concentration of aerosols is much lower than in the baseline scenario with no intervention.

Figure 5: It would be helpful to indicate the assumed reduction in transmission probability for each intervention directly in or alongside this figure, so that the reader can connect the parameter assumptions to the observed effect sizes.

Response: We now indicate the changes in parameter values in the Figure caption.

Fig 5. Impacts of control interventions on secondary attack rates, epidemic duration, and extinction probability across transmission schemes. (**A**) Relative difference of the 90-day cumulative incidence among patients and HCWs between the intervention scenario and the baseline scenario (with no intervention). In the hand hygiene scenario, the transmission rate between HCWs and patients was reduced by 14%. In scenarios with targeting masking, transmission rates among patients and between HCWs and patients was reduced by 70% and aerosol exhalation by 94%. In the universal masking scenario, transmission was reduced by 70% in all pairs of individuals, and aerosol exhalation by 94%. In scenarios with ventilation, we increased ACH to 8. Negative values indicate a reduction in the cumulative incidence when an intervention is in place. Relative difference estimates and error bars are derived from the pseudo-median and the 95% CI of Wilcoxon tests, respectively. Reductions stronger than 100% are explained by the definition of the metric: the median of the cumulative incidence in the baseline scenario used as the reference can be higher than the 95% CI bounds of the difference. (**B**) Distribution of epidemic durations in the baseline and intervention scenarios. (**C**) Extinction probability in the baseline and intervention scenarios.
Scheme 1: β_e = 1/45 new cases per infectious aerosol per day and β_c = 0.75 new case per contact per day; Scheme 2: β_e = 1/60 and β_c= 1.00; Scheme 3: β_e = 1/70 and β_c = 1.25; Scheme 4: β_e = 1/100 and β_c = 1.50; and Scheme 5: β_e = 1/150 and β_c = 1.75.

Have the authors made all data and (if applicable) computational code underlying the findings in their manuscript fully available?

The PLOS Data policy requires authors to make all data and code underlying the findings described in their manuscript fully available without restriction, with rare exception (please refer to the Data Availability Statement in the manuscript PDF file). The data and code should be provided as part of the manuscript or its supporting information, or deposited to a public repository. For example, in addition to summary statistics, the data points behind means, medians and variance measures should be available. If there are restrictions on publicly sharing data or code —e.g. participant privacy or use of data from a third party—those must be specified.

Reviewer #1: **No:** Interaction data from the Nods-Cov-2 project are not publicly available but can be made available from the corresponding author on reasonable request. All codes and synthetic contact data necessary to perform the simulation study are available online at https://github.com/MESuRS-Lab/aerosol-epi-model.

Reviewer #2: **No:** The code was made available but not the data. I recommend including synthetic data in the publication.

Response: We have updated the Github repository where we now provide synthetic data that have the same structure as the real data. We have modified the data availability statement accordingly.

Data availability statement: Interaction data from the Nods-Cov-2 project are not publicly available but can be made available from the Institut Pasteur (contact point: Prof Dider Guillemot) on reasonable request. Instead, we provide synthetic contact networks, and the corresponding staff and patient presence information that are available at https://github.com/MESuRS-Lab/aerosol-epi-model.

References

1. Duval A, Obadia T, Martinet L, Boëlle P-Y, Fleury E, Guillemot D, et al. Measuring dynamic social contacts in a rehabilitation hospital: effect of wards, patient and staff characteristics. Sci Rep. 2018;8: 1686. doi:10.1038/s41598-018-20008-w

2. Shirreff G, Huynh B-T, Duval A, Pereira LC, Annane D, Dinh A, et al. Assessing respiratory epidemic potential in French hospitals through collection of close contact data (April–June 2020). Sci Rep. 2024;14: 3702. doi:10.1038/s41598-023-50228-8

3. Crosby JC, Santos Leon E, Chinnock B, Harland KK, Krishnadasan A, Mohr NM, et al. Presenteeism Among Health Care Personnel With COVID-19. JAMA Netw Open. 2025;8: e2546405. doi:10.1001/jamanetworkopen.2025.46405

4. Madrazo L, Choo-Foo J, Yu W, LaDonna KA, Domecq M-C, Humphrey-Murto S. Going to work sick: A scoping review of illness presenteeism among physicians and medical trainees. Med Educ. 2025;59: 469–483. doi:10.1111/medu.15538

5. Foulkes S, Munro K, Sparkes D, Khawam J, Russell S, Dunne A, et al. Prevalence and impact of SARS-CoV-2, influenza, respiratory syncytial virus (RSV) infection and respiratory illness on UK healthcare workers during winter 2023/24 (September 2023 to March 2024): SIREN cohort study. J Infect. 2025;91. doi:10.1016/j.jinf.2025.106620

6. Temime L, Cohen N, Ait-Bouziad K, Denormandie P, Dab W, Hocine MN. Impact of a multicomponent hand hygiene-related intervention on the infectious risk in nursing homes: A cluster randomized trial. Am J Infect Control. 2018;46: 173–179. doi:10.1016/j.ajic.2017.08.030

7. Leung NHL. Transmissibility and transmission of respiratory viruses. Nat Rev Microbiol. 2021;19: 528–545. doi:10.1038/s41579-021-00535-6

8. Leung NHL, Milton DK. New WHO proposed terminology for respiratory pathogen transmission. Nat Rev Microbiol. 2024;22: 453–454. doi:10.1038/s41579-024-01067-5

9. Pöhlker ML, Pöhlker C, Krüger OO, Förster J-D, Berkemeier T, Elbert W, et al. Respiratory aerosols and droplets in the transmission of infectious diseases. Rev Mod Phys. 2023;95: 045001. doi:10.1103/RevModPhys.95.045001

10. Xiao S, Li Y, Sung M, Wei J, Yang Z. A study of the probable transmission routes of MERS-CoV during the first hospital outbreak in the Republic of Korea. Indoor Air. 2018;28: 51–63. doi:10.1111/ina.12430

11. Xiao S, Li Y, Wong T, Hui DSC. Role of fomites in SARS transmission during the largest hospital outbreak in Hong Kong. PLOS ONE. 2017;12: e0181558. doi:10.1371/journal.pone.0181558

12. Illingworth CJR, Yahiaoui G, Butler M, Brock RC, Peters C, Thaxter R, et al. The role of spatial distance in SARS-CoV-2 nosocomial transmission. J Hosp Infect. 2025;0. doi:10.1016/j.jhin.2025.09.011

13. Evans S, Stimson J, Pople D, Bhattacharya A, Hope R, White PJ, et al. Quantifying the contribution of pathways of nosocomial acquisition of COVID-19 in English hospitals. Int J Epidemiol. 2022;51: 393–403. doi:10.1093/ije/dyab241

14. Tsang T-W, Mui K-W, Wong L-T. Computational Fluid Dynamics (CFD) studies on airborne transmission in hospitals: A review on the research approaches and the challenges. J Build Eng. 2023;63: 105533. doi:10.1016/j.jobe.2022.105533

15. Ferrer J, Boelle P-Y, Salomon J, Miliani K, L'Hériteau F, Astagneau P, et al. Management of nurse shortage and its impact on pathogen dissemination in the intensive care unit. Epidemics. 2014;9: 62–69. doi:10.1016/j.epidem.2014.07.002

16. Oodally A, Hammami P, Reilhac A, Lamérie GG de, Opatowski L, Temime L. Hospital-level work organization drives the spread of SARS-CoV-2 within hospitals: insights from a multi-ward model. medRxiv; 2021. p. 2021.09.09.21262609. doi:10.1101/2021.09.09.21262609

17. English KM, Langley JM, McGeer A, Hupert N, Tellier R, Henry B, et al. Contact among healthcare workers in the hospital setting: developing the evidence base for innovative approaches to infection control. BMC Infect Dis. 2018;18: 184. doi:10.1186/s12879-018-3093-x

18. Whiteley TD, Stimson J, Brown CS, Robotham JV, Evans S. Modeling the impact of health care worker masking to reduce nosocomial SARS-CoV-2 transmission under varying adherence, prevalence, and transmission settings. Infect Control Hosp Epidemiol. 2025; 1–7. doi:10.1017/ice.2025.78

19. Bridgen JRE, Lewis JM, Todd S, Taegtmeyer M, Read JM, Jewell CP. A Bayesian approach to identifying the role of hospital structure and staff interactions in nosocomial transmission of SARS-CoV-2. J R Soc Interface. 2024;21: 20230525. doi:10.1098/rsif.2023.0525

20. Bansal A, Trieu M-C, Eriksson EM, Zhou F, McVernon J, Brokstad KA, et al. SARS-CoV-2 infection rates and associated risk factors in healthcare workers: systematic review and meta-analysis. Sci Rep. 2025;15: 4705. doi:10.1038/s41598-025-89472-5

21. Cocker D, Birgand G, Zhu N, Rodriguez-Manzano J, Ahmad R, Jambo K, et al. Healthcare as a driver, reservoir and amplifier of antimicrobial resistance: opportunities for interventions. Nat Rev Microbiol. 2024;22: 636–649. doi:10.1038/s41579-024-01076-4

22. Chan LYH, Morris SE, Stockwell MS, Bowman NM, Asturias E, Rao S, et al. Estimating the generation time for influenza transmission using household data in the United States. Epidemics. 2025;50: 100815. doi:10.1016/j.epidem.2025.100815

23. Zhang C, Huang X, Fang VJ, Chan K-H, Leung GM, Ip DKM, et al. Frequent presymptomatic household transmission of influenza A but not influenza B virus. Nat Health. 2026;1: 206–215. doi:10.1038/s44360-025-00049-y

24. Lessler J, Reich NG, Brookmeyer R, Perl TM, Nelson KE, Cummings DA. Incubation periods of acute respiratory viral infections: a systematic review. Lancet Infect Dis. 2009;9: 291–300. doi:10.1016/S1473-3099(09)70069-6

25. Yan J, Grantham M, Pantelic J, Bueno de Mesquita PJ, Albert B, Liu F, et al. Infectious virus in exhaled breath of symptomatic seasonal influenza cases from a college community. Proc Natl Acad Sci. 2018;115: 1081–1086. doi:10.1073/pnas.1716561115

26. Rockey NC, Le Sage V, Marr LC, Lakdawala SS. Seasonal influenza viruses decay more rapidly at intermediate humidity in droplets containing saliva compared to respiratory mucus. Appl Environ Microbiol. 90: e02010-23. doi:10.1128/aem.02010-23

27. Killingley B, Nguyen-Van-Tam J. Routes of influenza transmission. Influenza Other Respir Viruses. 2013;7: 42–51. doi:10.1111/irv.12080
