# Supplementary material for: Accounting for the long-distance transmission route: An epidemiological model of airborne disease transmission in hospitals
Source: PLoS Comput Biol. 2026 Aug 3;22(8):e1014564. doi: 10.1371/journal.pcbi.1014564 (PMC13460748; doi:10.1371/journal.pcbi.1014564)
Supplement: Supplementary file 3 [file pcbi.1014564.s003.docx]

**Peer Review**

*Gaufrès et al. “Accounting for the long-distance transmission route: an epidemiological model of airborne disease transmission in hospitals”*

# Summary

Thank you for the opportunity to review this paper.

This paper proposes a novel stochastic SEIR-type epidemiological model that integrates both short-range (close-contact) and long-range (aerosol) transmission routes of respiratory pathogens in hospital ICUs. The framework is applied to proximity sensor data from two French ICUs (Nods-Cov-2 study), using SARS-CoV-2 as a case study. The authors define five transmission “pathways” that vary the relative contribution of each route while maintaining similar overall epidemic intensity, and evaluate seven intervention scenarios including masking, hand hygiene, and ventilation.

The paper addresses an important gap: most nosocomial epidemiological models treat transmission as contact-driven, while biophysical and CFD models capture aerosol dynamics but over very short timescales and without population-level epidemic dynamics. Bridging these two traditions is valuable and timely, particularly in the post-COVID era. The framework of varying the relative contributions of short- vs. long-range routes and then evaluating tailored interventions is a practical and policy-relevant contribution. The location reconstruction algorithm derived from close-proximity sensor data is a useful methodological contribution for future studies.

However, I have several concerns regarding the clarity of the underlying hypothesis, the treatment of infected healthcare workers in the model, and the parameterisation of intervention scenarios which I detail below.

# Recommendation

I recommend **major revision**. The modelling framework is a valuable contribution, but the paper would benefit from: (1) a clearer articulation of the scientific question and the limitations of a scenario-based approach; (2) the inclusion of HCW isolation/removal as a realistic model feature; (3) a critical re-evaluation of the hand hygiene intervention parameterisation, which currently relies on community-setting evidence that is poorly suited to the model’s SARS-CoV-2 ICU application; (4) presentation of intervention effectiveness stratified by HCWs, not only patients; and (5) an expanded discussion of the inter-ward implications of HCW infection via the aerosol route.

# Major Comments

## 1. Clarify the underlying hypothesis and study framing

The specific scientific question this model is designed to answer is not clearly articulated. The paper appears to operate between several possible aims: (a) demonstrating that the relative contribution of short- vs. long-range transmission matters for choosing optimal interventions; (b) showing that long-range aerosol transmission plays a non-negligible role in nosocomial outbreaks; or (c) providing a framework that can identify which route dominates given appropriate data. The current framing conflates these, and the study design, which assumes different route contributions rather than inferring them from data, can only address (a). I would recommend that the authors explicitly state that this is a scenario-based simulation study and clarify what can and cannot be concluded from it. If the goal is ultimately to build an inferential framework, a discussion of what empirical data (e.g., genomic reconstruction, environmental sampling) would be needed to estimate the relative contributions of each route would strengthen the paper considerably.

## 2. Identifiability and the long-range aerosol route

The model’s long-range transmission route captures exposure to pathogen-laden aerosols accumulated in room air. However, the well-mixed box model per room cannot distinguish aerosol inhalation from other environmental contamination pathways (e.g., fomite transmission via contaminated surfaces). This is relevant because the interventions that address these mechanisms differ (ventilation vs. surface disinfection). A discussion of identifiability, specifically whether and how the aerosol route can be distinguished from environmental contamination given available data, would be valuable, particularly if this framework is to be used for intervention planning for other pathogens.

## 3. Treatment of infected HCWs: absence of isolation or workforce depletion

A significant concern is the apparent absence of any mechanism for removing infected HCWs from the workforce. The model appears to allow infectious HCWs to continue working, making contacts, and shedding aerosols throughout their infectious period. This has several consequences:

First, it inflates HCW-driven transmission. The reported secondary attack rates of 50 to 76% among HCWs likely reflect this unrealistic continued exposure. In practice, symptomatic HCWs would be identified and removed from the care environment, truncating transmission chains. Second, there is an internal inconsistency: the paper tests universal masking “from symptom onset of the index case,” implying awareness of who is infectious. If the model can identify symptom onset, it should also be able to implement HCW isolation or sick leave at that point. Third, the finding that the long-distance aerosol route disproportionately affects HCWs is one of the paper’s most interesting results, but its consequences are not fully explored. Infected HCWs removed from the workforce would reduce staffing ratios and potentially compromise patient care, a workforce depletion effect that could feed back into epidemic dynamics. Implementing HCW removal upon infection (with or without a detection delay) would be a relatively straightforward model extension and would allow the authors to explore system resilience as an outcome.

## 4. Outcome metrics and intervention evaluation

The authors evaluate intervention effectiveness using cumulative incidence among patients (Figure 5A). However, a key finding of this paper is that the long-distance aerosol route disproportionately affects HCWs. If this is the case, then evaluating ventilation effectiveness primarily through patient outcomes will underestimate its value. Supplementary Figure 7 presents results stratified by individual category, which partially addresses this concern. However, this stratification should be presented in the main text rather than relegated to the supplement, given its centrality to the paper’s narrative. Ideally, a composite metric that captures both direct health outcomes and system resilience (e.g., staffing capacity) would provide a more policy-relevant assessment of intervention value.

## 5. HCWs as inter-ward bridges: expand in discussion

The model is confined to a single ICU, but one of its most striking findings, that long-range aerosol transmission disproportionately infects HCWs, has implications that extend beyond the ward. HCWs are the most mobile population in hospitals: they rotate between wards, float to other units, visit break rooms, cafeterias, and meeting spaces. If the long-range route is what primarily infects HCWs, then infected HCWs become the bridge for inter-ward and inter-department dissemination. This connects naturally to a metapopulation perspective on hospitals as networks of wards linked by HCW movement. I would encourage the authors to expand on this in the discussion, as it represents both a limitation of the current single-ward model and a compelling direction for future work, and it follows directly from their own results.

## 6. Hand hygiene intervention: questionable parameterisation, relevance for SARS-CoV-2, and supporting citations

The parameterisation of the hand hygiene intervention raises several concerns. The authors model hand hygiene as a 17% reduction in the contact transmission probability from HCW to patient (β*c, HCW→patient*), citing Aiello et al. (2008), Jefferson et al. (2008), and Ross et al. (2023).

**Setting mismatch:** All three references study hand hygiene in community settings: households, schools, daycare centres, and low- and middle-income countries. None examine HCW hand hygiene in ICUs or hospitals. The transmission dynamics, baseline hygiene practices, pathogen exposure intensity, and contact patterns differ fundamentally between these settings.

**Outcome mismatch:** The cited studies use broad syndromic outcomes (“respiratory illness,” “ILI”) that bundle together many pathogens with different transmission routes (rhinoviruses, adenoviruses, RSV, influenza). The 17 to 21% reductions reported likely reflect a composite of reduced fomite transmission, self-inoculation, and behavioural co-interventions. Applying this estimate to a single mechanistic parameter (β*c*) implies the entire effect operates through direct contact transmission, which is almost certainly not the case.

**Pathogen mismatch:** The model is parameterised for SARS-CoV-2, for which there is now strong evidence that airborne transmission is the dominant route and fomite transmission plays a negligible role (Greenhalgh et al., *The Lancet* 2021; Meyerowitz et al., *Annals of Internal Medicine* 2021; Wee et al., *F1000Research* 2021). Hand hygiene is primarily effective against fomite-mediated transmission. The inclusion of hand hygiene as an intervention for SARS-CoV-2 contact transmission requires stronger justification. If the authors intend the model to be general to respiratory pathogens, they should acknowledge that the hand hygiene effect estimate is likely an overestimate for SARS-CoV-2 specifically. While the authors performed sensitivity analyses, the realistic focus should be on lower effectiveness rather than higher ones.

**Supporting citation.** Relatedly, the authors cite Evans et al. (2022) to support the claim that transient hand carriage in HCWs might be an important route for viruses. However, Evans et al. models an “indirect patient-to-patient” pathway that encompasses multiple mechanisms (HCWs as vectors, contaminated fomites) without isolating hand carriage as the specific driver. The evidence for hand-mediated transmission of respiratory viruses is considerably weaker than for bacterial pathogens (e.g., MRSA, VRE, *C. difficile*) or enteric viruses (e.g., norovirus), where hand contamination is well-documented and central to transmission. The authors should either cite more specific evidence for hand carriage of respiratory viruses or reframe this limitation.

# Minor Comments

## 7. Data availability and reproducibility

I understand that the close-proximity sensor data from the Nods-Cov-2 study cannot be made publicly available. However, to support reproducibility and allow other researchers to test or extend this framework, the authors should at minimum provide mock or synthetic data that preserves the structure and format of the input data without disclosing individual-level information.

## 8. Ventilation intervention contextualisation

The scenario of increasing ventilation to 8 ACH in patient-accessible rooms would benefit from contextualisation: what are typical baseline ACH values in French ICUs? What is the feasibility and cost of achieving 8 ACH? Is this consistent with current guidelines (e.g., WHO or French national standards)?

## 9. Generalisability beyond SARS-CoV-2

The authors state that the model is applicable to other airborne pathogens (RSV, influenza), but the parameterisation is entirely SARS-CoV-2-specific. The relative importance of aerosol vs. contact routes, latent periods, infectious periods, and aerosol stability vary substantially across pathogens. One advantage of this framework is its potential generalisability; however, demonstrating this, either with an additional pathogen parameterisation or by more carefully delineating which conclusions are SARS-CoV-2-specific vs. general, would strengthen the paper.

# Editorial Comments

## 10. Terminology: “pathways” vs. “scenarios”

The term “pathway” to describe different parameter combinations giving varying weight to the two transmission routes is potentially confusing, as “pathway” in epidemiology often refers to a mechanistic route of transmission. Since these represent hypothetical parameter configurations chosen via grid search, the term “scenario” would be more appropriate and less ambiguous.

## 11. Figure-specific comments

**Figure 2A:** Please expand the caption to explain what the dots represent. The figure is somewhat interpretable but would benefit from a more detailed description of how individual movements are visualised.

**Figure 5:** It would be helpful to indicate the assumed reduction in transmission probability for each intervention directly in or alongside this figure, so that the reader can connect the parameter assumptions to the observed effect sizes.
