# Supplementary material for: Accounting for the long-distance transmission route: An epidemiological model of airborne disease transmission in hospitals
Source: PLoS Comput Biol. 2026 Aug 3;22(8):e1014564. doi: 10.1371/journal.pcbi.1014564 (PMC13460748; doi:10.1371/journal.pcbi.1014564)
Supplement: Peer review history

# Peer review: Decision Letter 0

**DOI:** 10.1371/journal.pcbi.1014564.r001

**Authors:** Thomas Leitner, Edward Hill

23 Mar 2026

PCOMPBIOL-D-26-00130

Accounting for the long-distance transmission route: an epidemiological model of airborne disease transmission in hospitals

PLOS Computational Biology

Dear Dr. Layan,

Thank you for submitting your manuscript to PLOS Computational Biology. After careful consideration, we feel that it has merit but does not fully meet PLOS Computational Biology's publication criteria as it currently stands.

Therefore, we invite you to submit a revised version of the manuscript that addresses the points raised during the review process.

Please submit your revised manuscript by May 23 2026 11:59PM. If you will need more time than this to complete your revisions, please reply to this message or contact the journal office at ploscompbiol@plos.org. When you're ready to submit your revision, log on to https://www.editorialmanager.com/pcompbiol/ and select the 'Submissions Needing Revision' folder to locate your manuscript file.

Please include the following items when submitting your revised manuscript:

* A letter that responds to each point raised by the editor and reviewer(s). You should upload this letter as a separate file labeled 'Response to Reviewers'. This file does not need to include responses to formatting updates and technical items listed in the 'Journal Requirements' section below.

* A marked-up copy of your manuscript that highlights changes made to the original version. You should upload this as a separate file labeled 'Revised Manuscript with Track Changes'.

* An unmarked version of your revised paper without tracked changes. You should upload this as a separate file labeled 'Manuscript'.

If you would like to make changes to your financial disclosure, competing interests statement, or data availability statement, please make these updates within the submission form at the time of resubmission. Guidelines for resubmitting your figure files are available below the reviewer comments at the end of this letter

We look forward to receiving your revised manuscript.

Kind regards,

Edward M. Hill

Academic Editor

PLOS Computational Biology

Thomas Leitner

Section Editor

PLOS Computational Biology

**Journal Requirements:**

If the reviewer comments include a recommendation to cite specific previously published works, please review and evaluate these publications to determine whether they are relevant and should be cited. There is no requirement to cite these works unless the editor has indicated otherwise.

1) Please ensure that the CRediT author contributions listed for every co-author are completed accurately and in full.

At this stage, the following Authors/Authors require contributions: Julien Derdevet, Olivier Gaufrès, Solen Kernéis, Quentin J. Leclerc, Lulla Opatowski, George Shirreff, Laura Temime, and Maylis Layan. Please ensure that the full contributions of each author are acknowledged in the 'Add/Edit/Remove Authors' section of our submission form.

The list of CRediT author contributions may be found here: https://journals.plos.org/ploscompbiol/s/authorship#loc-author-contributions

2) Please upload all main figures as separate Figure files in .tif or .eps format. For more information about how to convert and format your figure files please see our guidelines:

https://journals.plos.org/ploscompbiol/s/figures

3) Some material included in your submission may be copyrighted. According to PLOS’s copyright policy, authors who use figures or other material (e.g., graphics, clipart, maps) from another author or copyright holder must demonstrate or obtain permission to publish this material under the Creative Commons Attribution 4.0 International (CC BY 4.0) License used by PLOS journals. Please closely review the details of PLOS’s copyright requirements here: PLOS Licenses and Copyright. If you need to request permissions from a copyright holder, you may use PLOS's Copyright Content Permission form.

Please respond directly to this email and provide any known details concerning your material's license terms and permissions required for reuse, even if you have not yet obtained copyright permissions or are unsure of your material's copyright compatibility. Once you have responded and addressed all other outstanding technical requirements, you may resubmit your manuscript within Editorial Manager.

Potential Copyright Issues:

i) Figure 1. Please confirm whether you drew the images / clip-art within the figure panels by hand. If you did not draw the images, please provide (a) a link to the source of the images or icons and their license / terms of use; or (b) written permission from the copyright holder to publish the images or icons under our CC BY 4.0 license. Alternatively, you may replace the images with open source alternatives. See these open source resources you may use to replace images / clip-art:

- https://commons.wikimedia.org

- https://openclipart.org/.

4) In the online submission form, you indicated that Interaction data from the Nods-Cov-2 project are not publicly available but can be made available from the corresponding author on reasonable request.. All PLOS journals now require all data underlying the findings described in their manuscript to be freely available to other researchers, either

1. In a public repository

2. Within the manuscript itself

3. Uploaded as supplementary information.

This policy applies to all data except where public deposition would breach compliance with the protocol approved by your research ethics board. If your data cannot be made publicly available for ethical or legal reasons (e.g., public availability would compromise patient privacy), please explain your reasons by return email and your exemption request will be escalated to the editor for approval. Your exemption request will be handled independently and will not hold up the peer review process, but will need to be resolved should your manuscript be accepted for publication. One of the Editorial team will then be in touch if there are any issues.

5) Please send a completed 'Competing Interests' statement, including any COIs declared by your co-authors. If you have no competing interests to declare, please state 'The authors have declared that no competing interests exist'. Otherwise please declare all competing interests beginning with the statement 'I have read the journal's policy and the authors of this manuscript have the following competing interests'

**Reviewers' comments:**

Reviewer's Responses to Questions

**Comments to the Authors:**

**Please note here if the review is uploaded as an attachment.**

Reviewer #1: This study uses an agent-based modeling framework to evaluate the roles of close-proximity interactions and aerosol-mediated transmission in healthcare-associated infections (HAIs) in ICUs. The authors use real-world proximity data to generate synthetic contact networks and simulate the transmission of a pathogen with characteristics similar to SARS-CoV-2. They further evaluate several interventions and rank their effectiveness under different transmission scenarios. Aerosol-mediated transmission poses a significant challenge for HAI control, so this study addresses an important and timely question. Overall, the technical approach is sound and the findings are clearly presented. Below are several comments and questions that I hope will help further improve the manuscript.

1. In the simulation design, pathogen spread in the ICU is treated as occurring within a closed system by introducing a single infection at the beginning of the simulation. This design overlooks an important component of HAI dynamics—the interaction with the community. This issue is particularly relevant for SARS-CoV-2, which continues to circulate widely in the general population. Healthcare workers (HCWs) interact with family members and other community contacts, and newly admitted patients may introduce infections into the ICU. It seems feasible for the model to incorporate such external introductions without major changes to the model structure.

2. The contact data were collected over a 36-hour period. This short sampling window may miss systematic temporal patterns in hospital operations, such as weekly variations in patient volume (e.g., lower patient numbers during weekends). It would be helpful to discuss whether this limitation could affect the representativeness of the simulated contact networks and the robustness of the results.

3. Some details about the simulation setup are unclear. In particular, what time step was used in the simulations (e.g., minutes, hours, or days)? Close contacts can vary substantially in duration. The choice of time step could influence the calculation of the force of infection. For example, if the model was integrated at an hourly time step, how were short contacts lasting only a few minutes (possibly occurring multiple times within an hour) accounted for? Clarification of this issue would help readers better understand the simulation framework.

4. Intervention parameters were selected from the literature. Masking was assumed to reduce transmission rates by 70%, which may be somewhat optimistic. Under this assumption, universal masking appears capable of controlling nearly all simulated outbreak scenarios. However, real-world experience suggests that masking alone has often been insufficient to fully control SARS-CoV-2 transmission. A sensitivity analysis using lower masking effectiveness would strengthen the robustness of the conclusions.

5. In the simulations, transmission rates were varied to generate different scenarios. It would be helpful to clarify how these transmission rate settings relate to the basic reproduction number (R₀). Providing a mapping between the model parameters and implied transmissibility would help readers better interpret the simulated scenarios.

6. In the generated contact networks, HCWs account for the majority of individuals. In real ICU settings, when HCWs are identified as infected, protocols typically remove them from patient care to reduce risk to patients. While such reactive interventions may be less relevant for pathogens with frequent asymptomatic carriage (e.g., antimicrobial-resistant bacteria), they are relevant for SARS-CoV-2. A discussion of how such policies might influence transmission dynamics in the modeled setting would be valuable.

7. The use of the term “long-range” transmission may not be entirely accurate in the context of the model. Aerosol transmission in the simulations appears to occur within the same room rather than over large spatial distances. Its main effect is to allow transmission among individuals who do not have direct spatiotemporal overlap in close contact. Clarifying the intended meaning of this term would improve precision.

Reviewer #2: Please find my review attached.

**********

**Have the authors made all data and (if applicable) computational code underlying the findings in their manuscript fully available?**

The PLOS Data policy requires authors to make all data and code underlying the findings described in their manuscript fully available without restriction, with rare exception (please refer to the Data Availability Statement in the manuscript PDF file). The data and code should be provided as part of the manuscript or its supporting information, or deposited to a public repository. For example, in addition to summary statistics, the data points behind means, medians and variance measures should be available. If there are restrictions on publicly sharing data or code —e.g. participant privacy or use of data from a third party—those must be specified.

Reviewer #1: **No:** Interaction data from the Nods-Cov-2 project are not publicly available but can bemade available from the corresponding author on reasonable request. All codes and synthetic contact data necessary to perform the simulation study are available online at https://github.com/MESuRS-Lab/aerosol-epi-model.

Reviewer #2: **No:** The code was made available but not the data. I recommend including synthetic data in the publication.

**********

PLOS authors have the option to publish the peer review history of their article (what does this mean?). If published, this will include your full peer review and any attached files.

If you choose “no”, your identity will remain anonymous but your review may still be made public.

**Do you want your identity to be public for this peer review?**  For information about this choice, including consent withdrawal, please see our Privacy Policy.

Reviewer #1: No

Reviewer #2: **Yes:** Thi Mui Pham

[NOTE: If reviewer comments were submitted as an attachment file, they will be attached to this email and accessible via the submission site. Please log into your account, locate the manuscript record, and check for the action link 'View Attachments'. If this link does not appear, there are no attachment files.]

**Figure resubmission:**

After uploading your figures to PLOS’s NAAS tool - https://ngplosjournals.pagemajik.ai/artanalysis, NAAS will process the files provided and display the results in the 'Uploaded Files' section of the page as the processing is complete. If the uploaded figures meet our requirements (or NAAS is able to fix the files to meet our requirements), the figure will be marked as 'fixed' above. If NAAS is unable to fix the files, a red 'failed' label will appear above. When NAAS has confirmed that the figure files meet our requirements, please download the file via the download option, and include these NAAS processed figure files when submitting your revised manuscript.

**Reproducibility:**

To enhance the reproducibility of your results, we recommend that authors of applicable studies deposit laboratory protocols in protocols.io, where a protocol can be assigned its own identifier (DOI) such that it can be cited independently in the future. Additionally, PLOS ONE offers an option to publish peer-reviewed clinical study protocols. Read more information on sharing protocols at https://plos.org/protocols?utm_medium=editorial-email&utm_source=authorletters&utm_campaign=protocols

Attachment Submitted filename: peer_review.docx

# Author comment: Author response to Decision Letter 1

**DOI:** 10.1371/journal.pcbi.1014564.r002

11 Jun 2026

Attachment Submitted filename: Response to reviewers.pdf

# Peer review: Decision Letter 1

**DOI:** 10.1371/journal.pcbi.1014564.r003

**Authors:** Thomas Leitner, Edward Hill

9 Jul 2026

Dear Miss Layan,

We are pleased to inform you that your manuscript 'Accounting for the long-distance transmission route: an epidemiological model of airborne disease transmission in hospitals' has been provisionally accepted for publication in PLOS Computational Biology.

Before your manuscript can be formally accepted you will need to complete some formatting changes, which you will receive in a follow up email. A member of our team will be in touch with a set of requests.

Please note that your manuscript will not be scheduled for publication until you have made the required changes, so a swift response is appreciated.

IMPORTANT: The editorial review process is now complete. PLOS will only permit corrections to spelling, formatting or significant scientific errors from this point onwards. Requests for major changes, or any which affect the scientific understanding of your work, will cause delays to the publication date of your manuscript.

Should you, your institution's press office or the journal office choose to press release your paper, you will automatically be opted out of early publication. We ask that you notify us now if you or your institution is planning to press release the article. All press must be co-ordinated with PLOS.

Thank you again for supporting Open Access publishing; we are looking forward to publishing your work in PLOS Computational Biology.

Best regards,

Edward M. Hill

Academic Editor

PLOS Computational Biology

Thomas Leitner

Section Editor

PLOS Computational Biology

***********************************************************

Reviewer's Responses to Questions

**Comments to the Authors:**

**Please note here if the review is uploaded as an attachment.**

Reviewer #1: My questions have been addressed in the revision.

Reviewer #2: The authors have addressed all of my concerns. I don't have any further comments.

**********

**Have the authors made all data and (if applicable) computational code underlying the findings in their manuscript fully available?**

The PLOS Data policy requires authors to make all data and code underlying the findings described in their manuscript fully available without restriction, with rare exception (please refer to the Data Availability Statement in the manuscript PDF file). The data and code should be provided as part of the manuscript or its supporting information, or deposited to a public repository. For example, in addition to summary statistics, the data points behind means, medians and variance measures should be available. If there are restrictions on publicly sharing data or code —e.g. participant privacy or use of data from a third party—those must be specified.

Reviewer #1: None

Reviewer #2: Yes

**********

PLOS authors have the option to publish the peer review history of their article (what does this mean?). If published, this will include your full peer review and any attached files.

If you choose “no”, your identity will remain anonymous but your review may still be made public.

**Do you want your identity to be public for this peer review?**  For information about this choice, including consent withdrawal, please see our Privacy Policy.

Reviewer #1: No

Reviewer #2: **Yes:** Thi Mui Pham

# Editor report: Acceptance letter

**DOI:** 10.1371/journal.pcbi.1014564.r004

**Authors:** Thomas Leitner, Edward Hill

PCOMPBIOL-D-26-00130R1

Accounting for the long-distance transmission route: an epidemiological model of airborne disease transmission in hospitals

Dear Dr Layan,

I am pleased to inform you that your manuscript has been formally accepted for publication in PLOS Computational Biology. Your manuscript is now with our production department and you will be notified of the publication date in due course.

The corresponding author will soon be receiving a typeset proof for review, to ensure errors have not been introduced during production. Please review the PDF proof of your manuscript carefully, as this is the last chance to correct any errors. Please note that major changes, or those which affect the scientific understanding of the work, will likely cause delays to the publication date of your manuscript.

Soon after your final files are uploaded, unless you have opted out, the early version of your manuscript will be published online. The date of the early version will be your article's publication date. The final article will be published to the same URL, and all versions of the paper will be accessible to readers.

For Research, Software, and Methods articles, you will receive an invoice from PLOS for your publication fee after your manuscript has reached the completed accept phase. If you receive an email requesting payment before acceptance or for any other service, this may be a phishing scheme. Learn how to identify phishing emails and protect your accounts at https://explore.plos.org/phishing.

Thank you again for supporting PLOS Computational Biology and open-access publishing. We are looking forward to publishing your work!

With kind regards,

Sharmila Kamatchi

PLOS Computational Biology | Carlyle House, Carlyle Road, Cambridge CB4 3DN | United Kingdom ploscompbiol@plos.org | Phone +44 (0) 1223-442824 | ploscompbiol.org | @PLOSCompBiol
